# The Partner Switching System of the SigF Sigma Factor in *Mycobacterium smegmatis* and Induction of the SigF Regulon Under Respiration-Inhibitory Conditions

**DOI:** 10.3389/fmicb.2020.588487

**Published:** 2020-11-11

**Authors:** Yuna Oh, Su-Yeon Song, Hye-Jun Kim, Gil Han, Jihwan Hwang, Ho-Young Kang, Jeong-Il Oh

**Affiliations:** Department of Integrated Biological Science, Pusan National University, Busan, South Korea

**Keywords:** *aa*_3_ cytochrome *c* oxidase, anti-sigma factor, anti-anti-sigma factor, electron transport chain, *Mycobacterium*, partner switching system, protein kinase, SigF

## Abstract

The partner switching system (PSS) of the SigF regulatory pathway in *Mycobacterium smegmatis* has been previously demonstrated to include the anti-sigma factor RsbW (MSMEG_1803) and two anti-sigma factor antagonists RsfA and RsfB. In this study, we further characterized two additional RsbW homologs and revealed the distinct roles of three RsbW homologs [RsbW1 (MSMEG_1803), RsbW2 (MSMEG_6129), and RsbW3 (MSMEG_1787)] in the SigF PSS. RsbW1 and RsbW2 serve as the anti-sigma factor of SigF and the protein kinase phosphorylating RsfB, respectively, while RsbW3 functions as an anti-SigF antagonist through its protein interaction with RsbW1. Using relevant mutant strains, RsfB was demonstrated to be the major anti-SigF antagonist in *M. smegmatis*. The phosphorylation state of Ser-63 was shown to determine the functionality of RsfB as an anti-SigF antagonist. RsbW2 was demonstrated to be the only protein kinase that phosphorylates RsfB in *M. smegmatis*. Phosphorylation of Ser-63 inactivates RsfB to render it unable to interact with RsbW1. Our comparative RNA sequencing analysis of the wild-type strain of *M. smegmatis* and its isogenic Δ*aa*_3_ mutant strain lacking the *aa*_3_ cytochrome *c* oxidase of the respiratory electron transport chain revealed that expression of the SigF regulon is strongly induced under respiration-inhibitory conditions in an RsfB-dependent way.

## Introduction

Sigma factors reversibly associate with the core RNA polymerase and function as specific factors that direct transcription of a specific subset of genes. 28 sigma factor genes were found to occur in *Mycobacterium smegmatis* in contrast to 13 genes in *Mycobacterium tuberculosis* (Cole et al., 1998; Manganelli et al., 2004; Waagmeester et al., 2005; Rodrigue et al., 2006). SigF belongs to group III sigma factors and is dispensable for growth of *M. tuberculosis* and *M. smegmatis* (Chen et al., 2000; Williams et al., 2007; Provvedi et al., 2008; Singh et al., 2015). SigF is phylogenetically and functionally in close relation to the well-studied stress sigma factor SigB in *Bacillus* species (DeMaio et al., 1996, 1997; Hecker and Volker, 2001; Hecker et al., 2007; Bouillet et al., 2018).

The *sigF* gene is widely conserved in mycobacterial species (Singh and Singh, 2008; Sachdeva et al., 2010). In *M. tuberculosis*, SigF (Rv3286c) was shown to be involved in virulence, biofilm formation, and diverse stress responses (Chen et al., 2000; Geiman et al., 2004; Karls et al., 2006; Williams et al., 2007; Hartkoorn et al., 2012; Manganelli, 2014). SigF (MSMEG_1804) in *M. smegmatis* was suggested to play roles in adaptation to stationary phase and conditions of heat and oxidative stress (Gebhard et al., 2008; Humpel et al., 2010; Singh et al., 2015). Overexpression or deletion of *sigF* was reported to alter cell wall architectures in *M. tuberculosis*, *Mycobacterium bovis*, and *M. smegmatis* (Forrellad et al., 2013; Singh et al., 2015; Dutta et al., 2019). The inactivation of *sigF* in *M. smegmatis* was shown to result in the loss of carotenoid (isorenieratene) pigmentation, accompanying increased susceptibility to hydrogen peroxide (Provvedi et al., 2008; Humpel et al., 2010; Singh et al., 2015). The consensus sequence (GGWWT-N_16__–__17_-GGGTAY) was suggested for the mycobacterial SigF-recognizing promoters (Gebhard et al., 2008; Provvedi et al., 2008; Humpel et al., 2010). The *sigF* genes of *M. tuberculosis* and *M. smegmatis* were demonstrated to be cotranscribed with their cognate anti-sigma factor genes *usfX* (*Rv3287c*) and *rsbW* (*MSMEG_1803*), respectively (DeMaio et al., 1997; Gebhard et al., 2008). Transcription of the *usfX-sigF* operon in *M. tuberculosis* is driven from a SigF-dependent promoter, whereas the *rsbW*-*sigF* operon in *M. smegmatis* is transcribed from two promoters, a SigF-independent promoter located immediately upstream of *rsbW* and a SigF-dependent promoter upstream of the *chaB* (*MSMEG_1802*) gene that is located 103 bp upstream of *rsbW* (Gebhard et al., 2008).

*M. tuberculosis sigF* was found to be strongly induced within cultured human macrophages, during stationary phase of growth, and upon exposure to cold shock, nutrient depletion, oxidative stress, and several antibiotics (rifampicin, ethambutol, streptomycin, and cycloserine), as well as in persister cells (DeMaio et al., 1996; Graham and Clark-Curtiss, 1999; Michele et al., 1999; Mariani et al., 2000; Betts et al., 2002; Keren et al., 2011; Forrellad et al., 2013), while *M. smegmatis sigF* was shown to be expressed at similar levels throughout its growth phase and only marginally increased under SigF-activating conditions (Gebhard et al., 2008; Singh and Singh, 2008).

**FIGURE 1:**
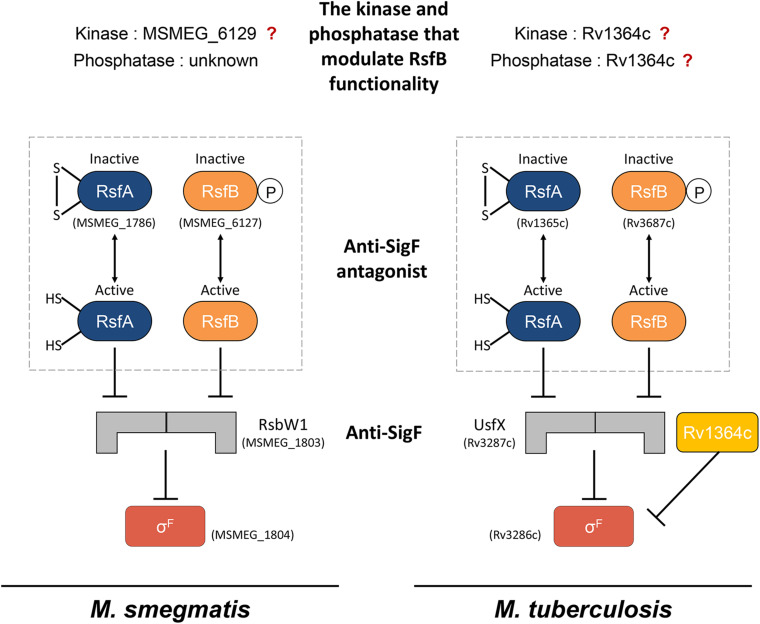
The schematic illustration outlining the current knowledge of the SigF PSS in *M. smegmatis* and *M. tuberculosis*. The “?” mark indicates the ambiguity of the roles of the corresponding proteins in the SigF PSS.

The functionality of SigF is regulated by the so-called partner switching system (PSS) including its cognate anti-sigma factor (anti-SigF) and anti-anti-sigma factors (anti-SigF antagonists) (Figure 1; DeMaio et al., 1997; Singh et al., 2015; Bouillet et al., 2018). Under non-stress (SigF-non-activating) conditions, SigF is held in an inactive state in complex with the anti-SigF (RsbW or UsfX). Under stress (SigF-activating) conditions, the release of SigF from its anti-SigF is accomplished by two anti-SigF antagonists, RsfA (Rv1365c in *M. tuberculosis* and MSMEG_1786 in *M. smegmatis*) and RsfB (Rv3687c in *M. tuberculosis* and MSMEG_6127 in *M. smegmatis*), which sequester the anti-SigF (Beaucher et al., 2002; Parida et al., 2005; Singh et al., 2015). RsfA is inactivated when a disulfide bond is formed between its two redox-responsive cysteine residues, while RsfB was suggested to be inactivated by phosphorylation (Beaucher et al., 2002; Manganelli et al., 2004). MSMEG_6129 was identified to be a protein kinase that phosphorylates RsfB in *M. smegmatis* (Bowman and Ghosh, 2014), while the kinase that is responsible for RsfB phosphorylation remains unknown in *M. tuberculosis*. Since an *MSMEG_6129* mutant strain of *M. smegmatis* paradoxically displayed decreased expression of the SigF regulon relative to the wild-type (WT) strain (Bowman and Ghosh, 2014), it was uncertain whether MSMEG_6129 is the kinase that inactivates RsfB by phosphorylation. Dephosphorylation of RsfB homologs in *Bacillus* species was demonstrated to be catalyzed by the PP2C family of phosphatases (Voelker et al., 1996; Vijay et al., 2000; Chen et al., 2003). However, no study has been published regarding which gene product is responsible for dephosphorylation of phosphorylated RsfB in mycobacteria. The *Rv1364c* gene in *M. tuberculosis* was found to encode a multi-domain protein consisting of the sensor, PP2C phosphatase, GHKL (gyrase, Hsp90, histidine kinase, MutL) kinase, and anti-sigma antagonist domains (Sachdeva et al., 2008; Greenstein et al., 2009; Misra et al., 2019). Rv1364c was shown to interact with SigF *in vitro*, suggesting the possibility that it might serve as an anti-SigF along with the major anti-SigF UsfX (Misra et al., 2019). Although Rv1364c was demonstrated to possess both kinase and phosphatase activities that autophosphorylate and autodephosphorylate its anti-sigma antagonist domain (Greenstein et al., 2009), the question remains unanswered regarding whether it can phosphorylate and dephosphorylate RsfB to modulate the anti-SigF antagonist activity of RsfB.

Using relevant mutant strains and protein interaction analyses, we here reveal the distinct roles of three RsbW homologs and two anti-SigF antagonists (RsfA and RsfB) in the SigF PSS of *M. smegmatis*. This study provides several lines of evidence showing that MSMEG_6129 is the only kinase in *M. smegmatis* that phosphorylates RsfB on Ser-63 to inactivate the functionality of RsfB as an anti-SigF antagonist. This study also presents the novel finding that expression of the SigF regulon in *M. smegmatis* is strongly induced under respiration-inhibitory conditions in an RsfB-dependent way.

## Materials and Methods

### Bacterial and Yeast Strains, Plasmids, and Culture Conditions

The bacterial and yeast strains and plasmids used in this study are listed in Table S1 in the supplementary material. *Escherichia coli* strains were cultivated in Luria-Bertani (LB) medium on a gyratory shaker (200 rpm) at 37°C. *M. smegmatis* strains were grown aerobically in Middlebrook 7H9 medium (Difco, Sparks, MD, United States) supplemented with 0.2% (w/v) glucose (7H9-glucose) and 0.02% (v/v) Tween 80 as an anti-clumping agent on a gyratory shaker at 37°C. Ampicillin (100 or 200 μg/ml for *E. coli*), kanamycin (50 μg/ml for *E. coli* and 15 or 30 μg/ml for *M. smegmatis*), chloramphenicol (34 μg/ml for *E. coli*) and hygromycin (200 μg/ml for *E. coli* and 50 μg/ml for *M. smegmatis*) were added to the growth medium when required. For treatment of *M. smegmatis* cultures with KCN, the cultures were grown to an optical density at 600 nm (OD_600_) of 0.45–0.5 and further incubated for 15 min following the addition of KCN to the cultures to a final concentration of 0.5 mM.

*Saccharomyces cerevisiae* strains were cultivated in YPD medium (Difco) or synthetic defined dropout (SD) medium (Clontech, Palo Alto, CA, United States) on a gyratory shaker at 30°C.

### DNA Manipulation and Transformation

Standard protocols and manufacturers’ instructions were followed for recombinant DNA manipulations (Green and Sambrook, 2012). Transformation of *M. smegmatis* and *S. cerevisiae* with plasmids was conducted by electroporation and the lithium acetate (LiAc)-mediated method, respectively, as previously described (Snapper et al., 1990; Guthrie and Fink, 1991).

### Site-Directed Mutagenesis

To introduce point mutations into the *rsfB* genes, PCR-based mutagenesis was performed using the Quick Change site-directed mutagenesis procedure (Stratagene, La Jolla, CA, United States). Synthetic oligonucleotides containing a mutated codon in the middle of their sequences were used to mutagenize the original codons. The primers used for mutagenesis are listed in Table S2. Mutations were verified by DNA sequencing.

### β-Galactosidase Assay and Determination of the Protein Concentration

β-Galactosidase activity in *M. smegmatis* was assayed spectrophotometrically as described elsewhere (Oh and Kaplan, 1999). Protein concentration was determined by using a Bio-Rad protein assay kit (Bio-Rad, Hercules, CA, United States) with bovine serum albumin as a standard protein.

### Reverse Transcription-PCR and Quantitative Real-Time PCR

RNA isolation from *M. smegmatis* strains, preparation of cDNA, reverse transcription PCR (RT-PCR), and quantitative real-time PCR (qRT-PCR) were conducted as described previously (Kim et al., 2010). The primers used for cDNA synthesis, RT-PCR, and qRT-PCR are listed in Table S2.

### Protein Purification

The C-terminally His_6_-tagged WT and mutant forms of RsfB were expressed in *E. coli* BL21 (DE3) strains harboring the pT7-7 derivative plasmids (pT7-7rsfB, pT7-7rsfBS63A, and pT7-7rsfBS63E). The strains harboring the pT7-7 derivatives were cultivated aerobically at 37°C in LB medium containing 100 μg/ml ampicillin to an OD_600_ of 0.4–0.6. Expression of the *rsfB* gene was induced by the addition of isopropyl-β-D-thiogalactopyranoside (IPTG) to a final concentration of 0.5 mM, and then cells were further grown for 4 h at 30°C. For purification of RsfB from *M. smegmatis*, the *M. smegmatis* strains containing pMHRsfB or pMHRsfBS63E were grown aerobically to an OD_600_ of 0.5–0.8 at 37°C in 7H9-glucose medium supplemented with 15 μg/ml kanamycin. Expression of the *rsfB* gene was induced by the addition of acetamide to a final concentration of 0.2% (w/v), and then cells were further grown for 7 h at 37°C. After 400 ml of *E. coli* or *M. smegmatis* cultures were harvested, cells were resuspended in 10 ml of buffer A [(20 mM Tris–HCl pH 8.0), 100 mM NaCl] containing 10 U/ml DNase I and 10 mM MgCl_2_. The resuspended cells were disrupted twice for *E. coli* or five times for *M. smegmatis* using a French pressure cell, and cell-free crude extracts were obtained by centrifugation twice at 14,000 × *g* for 15 min. 500 μl of 50% (v/v) slurry (bed volume: 250 μl) of Ni-Sepharose high performance resin (GE Healthcare, Piscataway, NJ, United States) was packed into a column. After equilibration of the resin with 10 bed volumes of buffer A, cell-free crude extracts were loaded into the column. The resin was washed with 40 bed volumes of buffer A containing 10 mM imidazole, 20 bed volumes of buffer A containing 30 mM imidazole, and then His_6_-tagged RsfB was eluted with 10 bed volumes of buffer A containing 100 mM imidazole. The eluted His_6_-tagged RsfB was diluted with buffer A to 10 mM imidazole and subjected to affinity chromatography again. The resin was washed with 20 bed volumes of buffer A containing 30 mM imidazole, and then His_6_-tagged RsfB was finally eluted with 10 bed volumes of buffer A containing 100 mM imidazole. Imidazole and NaCl were removed from purified RsfB by means of a PD-10 desalting column (GE Healthcare) equilibrated with 20 mM Tris–HCl (pH 8.0).

Purification of RsbW1 was conducted using *E. coli* BL21 (DE3) strains carrying pT7-7rsbW1. Cell-free crude extracts were loaded into the column packed with Ni-Sepharose resin. The resin was washed with 40 bed volumes of buffer A containing 10 mM imidazole, 20 bed volumes of buffer A containing 30 mM imidazole, and then His_6_-tagged RsfB was eluted with 10 bed volumes of buffer A containing 100 mM imidazole. RsbW2 and RsbW3 were purified from the *E. coli* BL21(DE3) strain carrying pT7-7rsbW2 and the *E. coli* Rosetta-gami 2 (DE3) strain with pT7-7rsbW3, respectively, in the same way as that of RsbW1 except for the modified wash and elution steps (40 bed volumes of buffer A containing 5 mM imidazole and 30 bed volumes of buffer A containing 50 mM imidazole for the wash step; 10 bed volumes of buffer A containing 250 mM imidazole for the elution step). SigF was purified from the *E. coli* BL21(DE3) strain carrying pT7-7sigF in the same way as RsbW2 except for the modified wash step (40 bed volumes of buffer A containing 10 mM imidazole and then 40 bed volumes of buffer A containing 25 mM imidazole).

### Western Blotting Analysis

To detect expressed SigF, RsbW1, and RsfB in cells, Western blotting analyses using rabbit polyclonal antibodies against the corresponding proteins were performed as described previously (Mouncey and Kaplan, 1998). For detection of His_6_-tagged proteins a mouse monoclonal antibody against His-3 (Santa Cruz Biotechnology, Santa Cruz, CA, United States; sc8036) was employed. The rabbit polyclonal antibodies and His-3 monoclonal antibody were used at a 1:20,000 and 1:2,000 dilution, respectively. To detect GroEL, a mouse monoclonal antibody against HSP65 (Santa Cruz Biotechnology; sc58170) was used at a 1:2,000 dilution. Alkaline phosphatase-conjugated anti-rabbit IgG produced in goat (Sigma, St. Louis, CA, United States; A0545) or alkaline phosphatase-conjugated anti-mouse IgG produced in rabbit (Sigma; A4312) was used at a 1:10,000 dilution for the detection of the primary antibodies.

### Analysis of *in vitro* Protein–Protein Interactions Using Non-denaturing PAGE

The mixture of two purified proteins in 20 mM Tris–HCl (pH 8.0) solution containing 20 mM β-mercaptoethanol was mixed with the same volume of 2× Binding buffer [40 mM Tris–HCl (pH 8.0), 0.01 mM EDTA (pH 8.0), 10 mM MgCl_2_, 20% (v/v) glycerol] and incubated for 25 min at room temperature. After the addition of 10× sample buffer [50 mM Tris–HCl (pH 6.8), 40% (w/v) sucrose, 0.05% (w/v) bromophenol blue], the mixtures were subjected to non-denaturing PAGE [7.5% (w/v) acrylamide] using electrophoresis buffer [2.5 mM Tris–HCl (pH 8.3), 19.2 mM glycine], which was initially run at 80 V for 1 h and subsequently at 100 V for 4 h. Non-denaturing PAGE was conducted at 4°C.

### Analysis of *in vivo* Protein–Protein Interactions Using Copurification Assay

To examine protein interactions of RsbW1 with RsbW2 and RsbW3 in *M. smegmatis*, copurification assay using Ni-Sepharose resin was performed. The C-terminally His_6_-tagged RsbW2 and RsbW3 were expressed in the WT strains of *M. smegmatis* harboring pMHRsbW2 and pMHRsbW3, respectively. The strains were grown aerobically to an OD_600_ of 0.45–0.5 at 37°C in 7H9-glucose medium supplemented with 15 μg/ml kanamycin and 0.1% (w/v) acetamide. After 200 ml of *M. smegmatis* cultures were harvested, cells were resuspended in 8 ml of buffer A containing 10 U/ml DNase I and 10 mM MgCl_2_. The resuspended cells were disrupted five times using a French pressure cell, and cell-free crude extracts were obtained by centrifugation twice at 14,000 × *g* for 15 min. 500 μl of the 50% (v/v) slurry (bed volume 250 μl) of Ni-Sepharose resin was packed into a column. After equilibration of the resin with 10 bed volumes of buffer A, cell-free crude extracts were loaded into the column. The resin was washed with 125 bed volumes of buffer A containing 10 mM imidazole and then His_6_-tagged RsbW2 and RsbW3 were eluted with 10 bed volumes of buffer A containing 250 mM imidazole. RsbW2 and RsbW3 in the eluents were detected by Western blotting analysis with a His-3 monoclonal antibody. The presence of RsbW1 in the eluents was determined using Western blotting analysis with RsbW1 polyclonal antibodies.

### Analysis of *in vivo* Protein–Protein Interactions Using Yeast Two-Hybrid Assay

*S. cerevisiae* AH109 strains cotransformed with both pGADT7linker and pGBKT7 derivatives were grown in SD medium (Clontech, Palo Alto, CA, United States) lacking leucine and tryptophan (SD/-Leu/-Trp). The overnight cultures were diluted with distilled water to an OD_600_ of 0.6 and spotted onto both solid SD/-Leu/-Trp plates and histidine-deficient SD/-Leu/-Trp/-His plates containing various concentrations of 3-amino-1,2,4-triazole (3-AT) for spotting assays. The plates were incubated at 30°C for 3–5 days.

### RNA Sequencing and Gene Expression Profiling

Three biological replicate cultures of the WT and Δ*aa*_3_ strains were grown aerobically to an OD_600_ of 0.45–0.5. Total RNA of each culture was isolated as described previously (Kim et al., 2010). rRNA was removed from the isolated total RNA using a Ribo-Zero rRNA Removal Kit (Bacteria) (Illumina, San Diego, CA, United States). The RNA sequencing libraries were created using a TruSeq RNA Sample Prep Kit v2 (Illumina) with the standard low-throughput protocol. Sequencing of the six libraries was conducted on an Illumina HiSeq 4000 platform at Macrogen Inc. (Seoul, South Korea) using the HiSeq 3000–4000 sequencing protocol and TruSeq 3000–4000 SBS Kit v3 reagent (Illumina). Paired-end reads (101 bp) were then mapped to the reference genome sequence of *M. smegmatis* mc^2^155 (GCF_000015005.1_ASM1500v1) with the program Bowtie 1.1.2 using default settings. Summarized statistics of RNA sequencing alignment are listed in Table S3. The differentially expressed genes (DEGs) were subsequently identified pair-wise by the edgeR package in R language (Robinson et al., 2010). In this analysis, the genes with *P*-value < 0.05 and | log_2_ fold change of gene expression (FC)| > 2 were regarded as DEGs. The data described in this study have been deposited in NCBI’s Gene Expression Omnibus and are accessible through the GEO Series accession number GSE155251.

### *In vitro* Kinase Assay

Purified RsfB was mixed with purified RsbW1, RsbW2, or RsbW3 in 30 μl of reaction buffer [20 mM Tris–Cl (pH 7.5), 50 mM NaCl, 10 mM MgCl_2_, and 10 mM MnCl_2_]. The reactions were started by adding 100 μM ATP and incubated for 30 min at 30°C. The reactions were terminated by adding 7.5 μl of gel-loading buffer [250 mM Tris–Cl (pH 6.8), 50% (w/v) glycerol, 500 mM dithiothreitol (DTT), 10% (w/v) SDS, 5% (v/v) β-mercaptoethanol, and 0.5% (w/v) bromophenol blue]. Proteins were resolved by Phos-tag SDS-PAGE prepared as described elsewhere (Barbieri and Stock, 2008). The duplicated reaction mixtures were subjected to normal SDS-PAGE. The gels were stained with Coomassie brilliant blue (CBB).

## Results

### Induction of the SigF Regulon Under Respiration-Inhibitory Conditions and the Genetic Organization of the Genes Involved in the SigF PSS

**FIGURE 2:**
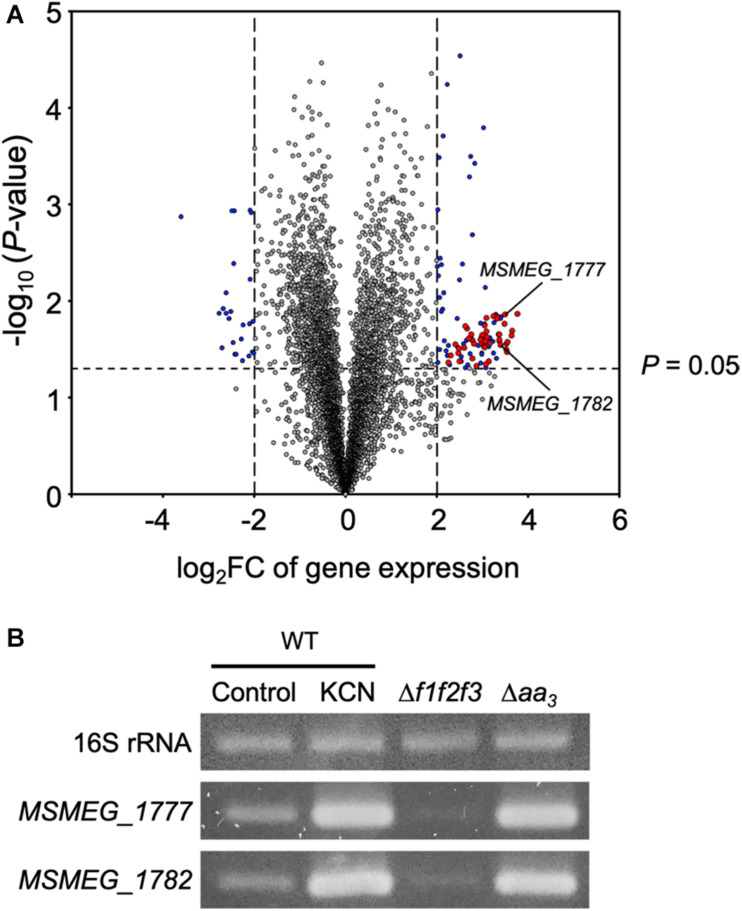
Overlap between the SigF regulon and the genes induced in the Δ*aa*_3_ mutant strain of *M. smegmatis*. **(A)** Volcano plot showing the DEGs in the Δ*aa*_3_ mutant strain relative to the WT strain. RNA sequencing was carried out using RNA extracted from three biological replicate cultures of the WT and Δ*aa*_3_ strains aerobically grown to an OD_600_ of 0.45–0.5 in 7H9-glucose medium. The *x*-axis represents log_2_ fold change of gene expression (log_2_FC) in the Δ*aa*_3_ mutant strain relative to the WT strain, and the y-axis represents –log_10_ (*P*-value). The vertical dashed lines on the graph mark the border lines indicating the log_2_FC values of –2 and 2. The genes, which are differently regulated by more than | log_2_FC| > 2 with *P*-value < 0.05, are depicted by blue-filled circles. Among the identified DEGs, the genes belonging to the SigF regulon are denoted by red-filled circles. The reported downregulated genes (log_2_FC < –2) in a *sigF* mutant strain relative to the WT strain were regarded as the genes belonging to the SigF regulon (Singh et al., 2015). Two representative genes (*MSMEG_1777* and *MSMEG_1782*), whose expression was confirmed by RT-PCR, are indicated by the arrows. **(B)** RT-PCR analysis showing the expression levels of the *MSMEG_1777* and *MSMEG_1782* in the WT, Δ*f1f2f3*, and Δ*aa*_3_ mutant strains. The *M. smegmatis* strains were grown aerobically to an OD_600_ of 0.45–0.5 in 7H9-glucose medium. For treatment of *M. smegmatis* cultures with KCN, the WT strain was grown to an OD_600_ of 0.45–0.5, followed by the addition of KCN to the final concentration of 0.5 mM. The WT cultures were further grown under KCN-untreated (control) or treated (0.5 mM KCN) conditions for 15 min. RT-PCR for 16S rRNA was performed to assure that equal amounts of total RNA were employed for RT-PCR.

Comparative RNA sequencing analysis of the WT strain of *M. smegmatis* and its isogenic Δ*aa*_3_ mutant strain with a deletion in *ctaC* encoding subunit III of the *aa*_3_ cytochrome *c* oxidase led us to identify 103 DEGs whose expression is upregulated in the Δ*aa*_3_ mutant by more than four-fold with a *P*-value less than 0.05 relative to the WT strain. As shown in Figure 2A and Table S4, 61 genes of the 103 DEGs were found to overlap with the genes belonging to the known SigF regulon (Singh et al., 2015), suggesting that the genes of the SigF regulon are strongly upregulated in *M. smegmatis*, when the major terminal oxidase of the electron transport chain (ETC) is inactivated. Among the 61 identified genes, we selected two genes (*MSMEG_1777* and *MSMEG_1782*) with the large FC and RPKM (reads per kilo base pair per million mapped reads) values in the Δ*aa*_3_ mutant, and examined the expression levels of the genes in the WT (control) and Δ*aa*_3_ mutant strains grown aerobically, as well as in the WT strain treated with KCN, an inhibitor of the *aa*_3_ cytochrome *c* oxidase (Figure 2B). Consistent with the RNA sequencing result, expression of *MSMEG_1777* and *MSMEG_1782* was significantly increased in the Δ*aa*_3_ mutant and the WT strain treated with KCN as compared to that in the control WT strain. We also included the Δ*f1f2f3* mutant strain of *M. smegmatis* with deletions in three *furA* paralogous genes in this experiment, since it had been reported that the genes of the SigF regulon are strongly downregulated in the Δ*f1f2f3* mutant relative to the WT strain (Lee et al., 2018). As expected, expression of *MSMEG_1777* and *MSMEG_1782* was significantly decreased in the mutant, confirming the *MSMEG_1777* and *MSMEG_1782* genes belong to the SigF regulon. Based on this result, we hereafter used the *MSMEG_1777* gene as a marker gene of the SigF regulon to determine the functionality of SigF.

**FIGURE 3:**
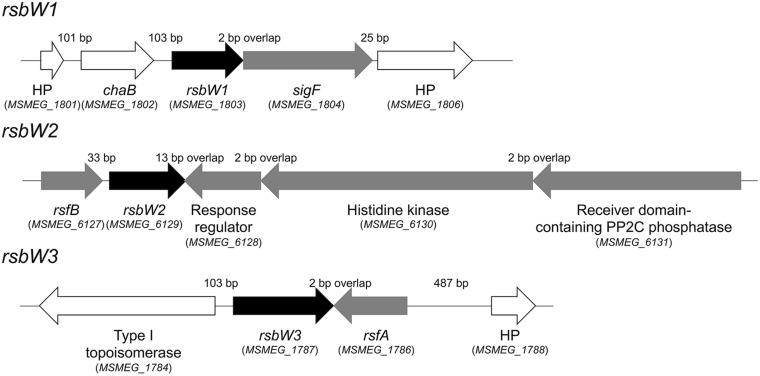
Genetic organization of the *rsbW1*, *rsbW2*, and *rsbW3* loci of *M. smegmatis* mc^2^ 155. The arrows indicate the open reading frames and their transcriptional direction. The locus tag numbers of the genes are presented in parentheses below the gene names. The lengths of the intergenic and overlapping regions are given as the nucleotide numbers above the schematic diagrams. The genes encoding hypothetical proteins are denoted by HP.

As a first step to understand the mechanism underlying the strong induction of the SigF regulon under respiration-inhibitory conditions, we decided to investigate the SigF PSS in detail. The *rsbW*-*sigF* (*MSMEG_1803*-*MSMEG_1804*) operon has been previously identified, and the role of RsbW (MSMEG_1803) as an anti-SigF in *M. smegmatis* has been suggested on the basis of its overexpression phenotype and its protein interaction with SigF (Singh et al., 2015). The genes encoding the proposed anti-SigF antagonists RsfA and RsfB have been also previously identified (Singh et al., 2015). To identify the additional genes whose products are likely to be involved in the SigF PSS, a BLAST search using the RsbW (MSMEG_1803) sequence as a query sequence was performed against the *M. smegmatis* mc^2^155 genome, which revealed two additional genes (*MSMEG_1787* and *MSMEG_6129*) that encode the RsbW homologs. As shown in Figure 3, The *MSMEG_1787* and *MSMEG_6129* genes were found to be located in the vicinity of the *rsfA* and *rsfB* genes, respectively. The RsbW homologs, whose genes are adjacent to *sigF*, *rsfB*, and *rsfA*, were named as RsbW1, RsbW2, and RsbW3, respectively. Among the three RsbW homologs, RsbW1 composed of 138 amino acids shows the highest homology (66.2% identity) to UsfX of *M. tuberculosis*. The *rsbW2* gene, whose product consists of 148 amino acids, appears to form an operon with the upstream gene *rsfB*. Downstream of *rsbW2* occurs a putative operon that contains the gene (*MSMEG_6130*) encoding a histidine kinase and two adjacent genes (*MSMEG_6128* and *MSMEG_6131*) encoding N-terminally receiver domain-containing proteins. Sequence analysis revealed that MSMEG_6128 and MSMEG_6131 contain a DNA-binding domain and a PP2C phosphatase domain at their C-termini, respectively. RsbW2 was previously reported to encode the protein kinase that phosphorylates the anti-SigF antagonist RsfB (Bowman and Ghosh, 2014). However, its inactivation by mutation was reported not to produce the anticipated phenotype like an increase in expression of the SigF regulon (Bowman and Ghosh, 2014), casting doubt as to whether RsbW2 is the kinase that inactivates RsfB by phosphorylation. The *rsbW3* gene was found to code for the largest RsbW homolog composed of 194 amino acids. When the amino acid sequence of RsbW3 was aligned with that of RsbW1, RsbW3 was found to have an N-terminal extension of 30 amino acids which is not present in RsbW1 (Supplementary Figure S1).

### The Roles of Three RsbW Homologs in the Regulation of SigF Functionality

**FIGURE 4:**
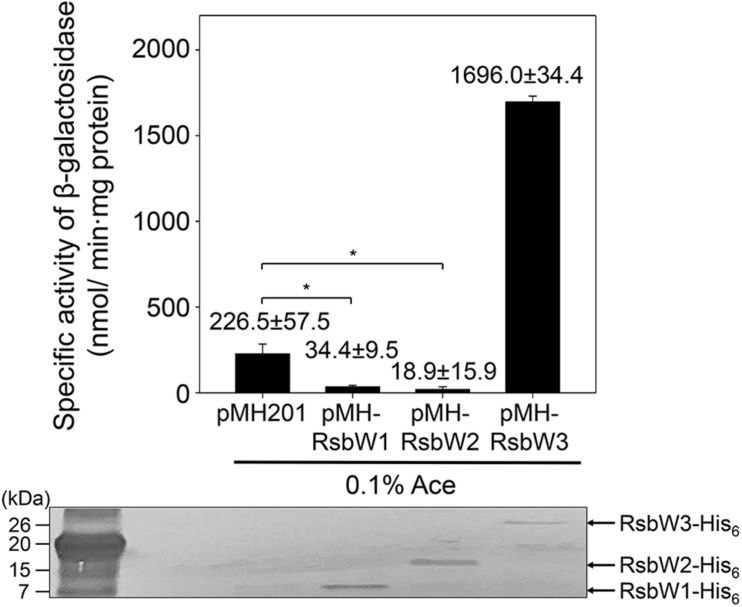
Overexpression effects of *rsbW1*, *rsbW2*, and *rsbW3* on *MSMEG_1777* expression. The *rsbW1*, *rsbW2*, and *rsbW3* genes were overexpressed from the pMH201 derivatives pMHRsbW1, pMHRsbW2, and pMHRsbW3, respectively. The overexpression effects of *rsbW1*, *rsbW2*, and *rsbW3* on the expression level of *MSMEG_1777* were determined in the *M. smegmatis* strains harboring the *MSMEG_1777*:*lacZ* translational fusion plasmid (pNCII1777) and the pMH201 derivatives. As a control, the *M. smegmatis* strain harboring both pNCII1777 and the empty pMH201 vector was employed in the experiment. The promotor activity of *MSMEG_1777* was measured by determining β-galactosidase activity in the strains grown aerobically to an OD_600_ of 0.45–0.5 in 7H9-glucose medium in the presence of 0.1% (w/v) acetamide (Ace). All values are the means of the results from three biological replicates. The error bars indicate the standard deviations. Western blotting analysis was conducted for the detection of expressed His_6_-tagged RsbW1, RsbW2, and RsbW3. Cell-free crude extracts (30 μg) were separated on SDS-PAGE, followed by Western blotting analysis with a His-tag antibody. *P < 0.05.

To examine whether the identified RsbW homologs function as anti-SigF, we individually inactivated the *rsbW1*, *rsbW2*, and *rsbW3* genes by deleting the corresponding genes, yielding the Δ*rsbW1*, Δ*rsbW2*, and Δ*rsbW3* mutant strains of *M. smegmatis* (Supplementary Figure S2). As shown in Supplementary Figure S3, both Δ*rsbW1* and Δ*rsbW2* mutants formed yellow-colored colonies on solid agar plates unlike the WT, Δ*rsbW3*, and Δ*sigF* strains of *M. smegmatis*, implying that biosynthesis of the carotenoid isorenieratene is increased in the Δ*rsbW1* and Δ*rsbW2* mutant strains. Given the previous report that the genes involved in biosynthesis of isorenieratene belong to the SigF regulon in *M. smegmatis* (Provvedi et al., 2008; Humpel et al., 2010), it is likely that expression of the SigF regulon is increased in the Δ*rsbW1* and Δ*rsbW2* mutants. Unfortunately, the Δ*rsbW1* and Δ*rsbW2* mutants were found to be instable in terms of yellow pigmentation. They lost the yellow color during cultivation in liquid growth medium, and the altered strains did not restore the yellow pigmentation in solid growth plates. For this reason, we did not examine expression of the SigF-dependent genes in the mutants. Instead, overexpression effects of *rsbW1*, *rsbW2*, and *rsbW3* on *MSMEG_1777* expression were examined to assess the anti-SigF activity of the three RsbW homologs. The genes of the RsbW homologs were overexpressed from an acetamide-inducible promoter on pMHRsbW1, pMHRsbW2, and pMHRsbW3 that are derivatives of the pMH201 integration vector. The expression level of *MSMEG_1777* in *M. smegmatis* was determined using an *MSMEG_1777*:*lacZ* translational fusion, pNCII1777. As shown in Figure 4, overexpression of *rsbW1* or *rsbW2* led to a significant decrease in *MSMEG_1777* expression in *M. smegmatis* compared to the control strain with pNCII1777 and pMH201, while overexpression of *rsbW3* rather strongly increased the expression of *MSMEG_1777*. Overexpression of *rsbW1*, *rsbW2*, and *rsbW3* in the *M. smegmatis* strains was verified by Western blotting analysis using a His-tag antibody. Altogether, the results indicate that overexpression of *rsbW1* and *rsbW2* inhibits the transcriptional activity of SigF, while that of *rsbW3* increases the functionality of SigF.

**FIGURE 5:**
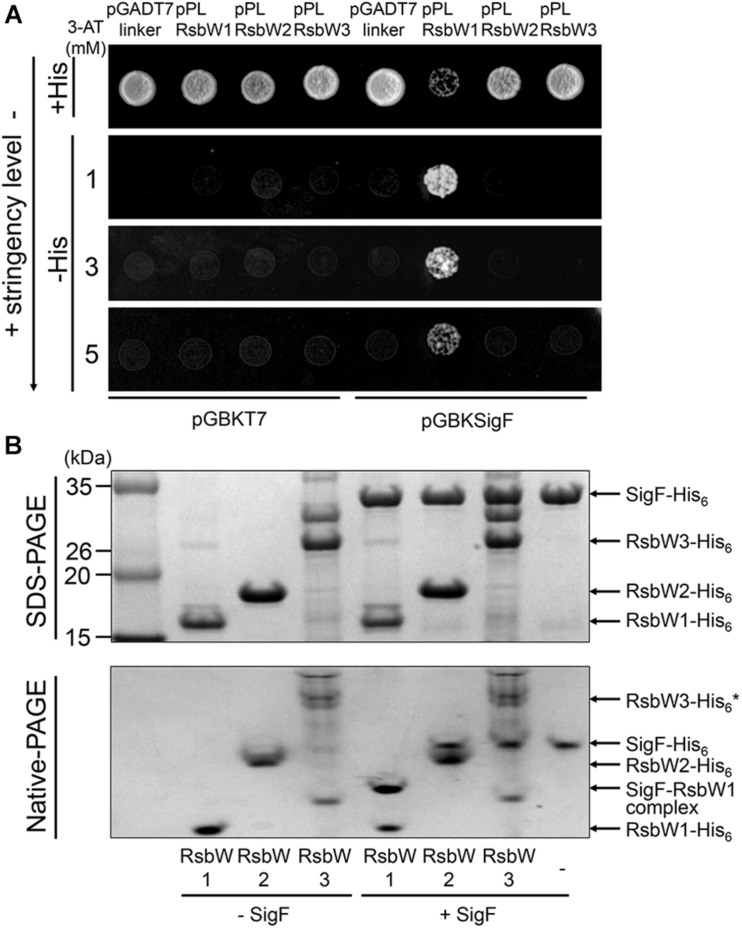
Determination of protein–protein interactions between SigF and RsbW homologs using Y2H assay and non-denaturing PAGE. **(A)** Y2H assay. The *sigF* gene is cloned into pGBKT7 (encoding the GAL4 DNA-binding domain), yielding pGBKSigF. The *rsbW1*, *rsbW2*, *rsbW3* genes were cloned into the pGADT7linker (encoding the GAL4 activation domain), resulting in pPLRsbW1, pPLRsbW2, and pPLRsbW3, respectively. The yeast strains cotransformed with both pGBKSigF and pGADT7linker derivative plasmids were employed for Y2H assay. To discriminate false positive interactions, the yeast strains with empty pGBKT7 and either pGADT7linker or the pGADT7linker derivatives were included in Y2H assay as negative controls. All yeast strains were spotted onto SD/-Leu/-Trp plates (+His) and histidine-deficient SD/-Leu/-Trp/-His plates (–His) containing different concentrations of 3-AT. The numbers to the left indicate the concentration of 3-AT. **(B)** Non-denaturing PAGE analysis. 100 pmol of purified SigF was mixed with purified RsbW1 (200 pmol), RsbW2 (200 pmol), or RsbW3 in binding buffer and incubated for 30 min at 25°C (+SigF). The mixtures were subjected to both SDS-PAGE (upper panel) and native PAGE (lower panel). As controls, the binding mixtures without the addition of SigF, which contain the RsbW homologs alone, were included in the experiment (–SigF). The gels were stained with CBB. The bands representing the RsbW homologs, SigF, and the RsbW1-SigF complex are indicated by the arrows. * Since RsbW3 was not purified to homogeneity, the RsbW3 band in the native PAGE gel is uncertain.

The prerequisite for a protein to act as an anti-sigma factor is protein–protein interactions between the protein and its cognate sigma factor. To determine protein–protein interactions between the RsbW homologs and SigF, we performed yeast two-hybrid assay (Y2H). For the Y2H assay, the *rsbW1*, *rsbW2*, and *rsbW3* genes were cloned into the prey vector pGADT7linker, whereas the *sigF* gene was cloned into the bait vector pGBKT7. As shown in Figure 5A, the yeast strain coexpressing RsbW1 and SigF grew well on solid growth medium without histidine (−His) in the presence of up to 5 mM 3-AT. In contrast, coexpression of either RsbW2 or RsbW3 with SigF did not lead to growth of yeast on −His medium in the presence of 3-AT. As expected, the yeast strains coexpressing either RsbW1 or SigF alone did not grow on −His medium in the presence of 3-AT. We next examined *in vitro* protein–protein interactions between SigF and RsbW homologs by means of non-denaturing PAGE analysis using purified SigF and RsbW homologs. Since the RsbW3 protein was not purified to homogeneity, the partially purified protein was used in the experiment. As shown in Figure 5B, RsbW1 was shown to interact with SigF as judged by the formation of a new band representing the SigF-RsbW1 complex and disappearance of the SigF band in the non-denaturing PAGE gel. In contrast, the presence of RsbW2 and RsbW3 in the binding mixtures did not result in a decrease in the SigF band intensity in the non-denaturing PAGE gel, indicating that RsbW2 and RsbW3 do not interact with SigF. Taken together, the Y2H and non-denaturing PAGE results suggest that SigF interacts only with RsbW1 among the three RsbW homologs.

**FIGURE 6:**
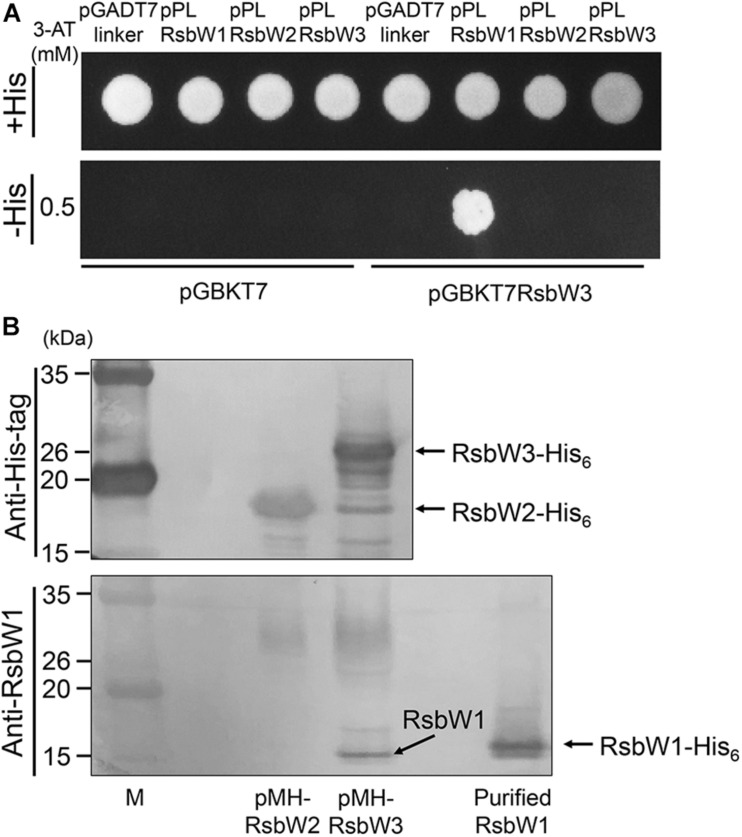
Determination of protein–protein interactions between RsbW3 and RsbW homologs using Y2H and copurification assays. **(A)** Y2H assay. The yeast strains carrying both pGBKT7-derived pGBKRsbW3 and pGADT7linker-derived plasmids (pPLRsbW1, pPLRsbW2, and pPLRsbW3) were employed for Y2H assay. The yeast strains harboring the empty pGBKT7 or pGADT7linker vector were used as negative controls. All yeast strains were spotted onto SD/-Leu/-Trp plates (+His) and histidine-deficient SD/-Leu/-Trp/-His plates (–His) containing 0.5 mM 3-AT. **(B)** Copurification assay. His_6_-tagged RsbW2 and RsbW3 were expressed in the WT strains of *M. smegmatis* with pMHRsbW2 and pMHRsbW3, respectively, and partially purified from their crude extracts by means of affinity chromatography using Ni-Sepharose resin. The purified proteins were subjected to Western blotting analyses using a His-3 monoclonal antibody for detection of His_6_-tagged RsbW2 and RsbW3 (upper panel), as well as RsbW1 polyclonal antibodies for detection of RsbW1 (lower panel). The bands representing RsbW1 and His_6_-tagged RsbW1, RsbW2, and RsbW3 are indicated by the arrows. M, molecular weight marker lanes.

The observed overexpression effect of *rsbW3* on *MSMEG_1777* led us to assume that RsbW3 might serve as an anti-SigF antagonist. To examine this assumption, protein–protein interactions between RsbW3 and three RsbW homologs were assessed using Y2H analysis (Figure 6A). For the Y2H assay, the *rsbW1*, *rsbW2*, and *rsbW3* genes were cloned into pGADT7linker, and the *rsbW3* gene was cloned into pGBKT7. Only the yeast strain coexpressing RsbW1 and RsbW3 grew on −His medium containing 0.5 mM 3-AT, indicating a possible protein interaction between RsbW1 and RsbW3. Protein–protein interactions between RsbW1 and RsbW3 were also assessed by copurification analysis using affinity chromatography (Figure 6B). RsbW1 was copurified with His_6_-tagged RsbW3 from crude extracts of the WT strain of *M. smegmatis* expressing His_6_-tagged RsbW3, whereas RsbW1 was not copurified with His_6_-tagged RsbW2 from the *M. smegmatis* strain expressing His_6_-tagged RsbW2, confirming protein–protein interactions between RsbW1 and RsbW3.

### Both RsfA and RsfB Are Functional as Anti-SigF Antagonists, and RsfB Is the Major Anti-SigF Antagonist in *M. smegmatis*

**FIGURE 7:**
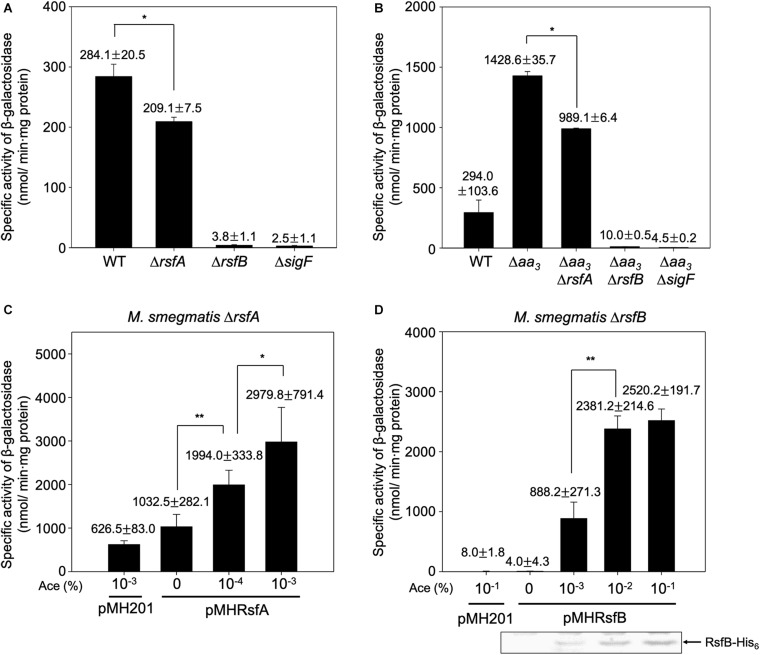
Effects of inactivation and overexpression of *rsfA* and *rsfB* on expression of *MSMEG_1777* in *M. smegmatis*. The expression level of *MSMRG_1777* was determined in the WT, Δ*rsfA*, Δ*rsfB*, Δ*sigF*, Δ*aa*_3_, Δ*aa*_3_Δ*rsfA*, Δ*aa*_3_Δ*rsfB*, and Δ*aa*_3_Δ*sigF* strains of *M. smegmatis* harboring pNCII1777 **(A,B)**. The *M. smegmatis* strains were grown aerobically to an OD_600_ of 0.45–0.5 in 7H9 medium. Cell-free crude extracts were used to measure β-galactosidase activity. All values are the means of the results from three biological replicates. The error bars indicate the standard deviations. *P < 0.05. Effects of increasing expression of *rsfA* and *rsfB* on *MSMEG_1777* expression were examined in the Δ*rsfA* and Δ*rsfB* strains harboring pNCII1777, respectively **(C,D)**. The pMH201-derived pMHRsfA plasmid was used for the controllable expression of *rsfA* in the Δ*rsfA* strain, and pMHRsfB was used for the controllable expression of *rsfB* in the Δ*rsfB* strain. The strains were grown aerobically to an OD_600_ of 0.45–0.5 in 7H9 medium supplemented with the indicated concentrations of acetamide (Ace). Cell-free crude extracts were used to measure β-galactosidase activity. All values are the means of the results from three biological replicates. The error bars indicate the standard deviations. Western blotting analysis was performed for the detection of expressed His_6_-tagged RsfB. Cell-free crude extracts (30 μg) were separated on SDS-PAGE, followed by Western blotting analysis with a His-tag antibody. **P < 0.01 and *P < 0.05.

After having established the distinct roles of three RsbW homologs in the SigF PSS, we next examined the roles of the suggested anti-SigF antagonists RsfA and RsfB *in vivo*. We constructed deletion mutants of *rsfA* and *rsfB* in the background of both WT and Δ*aa*_3_ strains, and the expression level of *MSMEG_1777* in the WT and mutant strains of *M. smegmatis* was comparatively determined (Figures 7A,B). The inactivation of the *aa*_3_ cytochrome *c* oxidase was used for an induction condition of the SigF regulon. The expression level of *MSMEG_1777* was shown to be increased by 4.9-fold in the Δ*aa*_3_ strain of *M. smegmatis* relative to that in the WT strain grown under the same conditions. Expression of *MSMEG_1777* was abolished in the Δ*sigF* and Δ*aa*_3_Δ*sigF* mutant strains. Both the results confirmed that transcription of *MSMEG_1777* depends on SigF, and that expression of the SigF regulon is induced under respiration-inhibitory conditions. Expression of *MSMEG_1777* was decreased by 26% in the Δ*rsfA* mutant compared to the WT strain. The Δ*aa*_3_Δ*rsfA* mutant also showed a 31% decrease in *MSMEG_1777* expression relative to the Δ*aa*_3_ mutant strain. It is noteworthy that inactivation of *rsfB* almost abolished expression of *MSMEG_1777* in both WT and Δ*aa*_3_ mutant strains. These results suggest that both RsfA and RsfB serve as anti-SigF antagonists, and that RsfB is the major anti-SigF antagonist in *M. smegmatis* under both SigF-activating and SigF-non-activating conditions. To confirm the roles of RsfA and RsfB as anti-SigF antagonists, we examined whether *MSMEG_1777* expression correlates with the expression levels of RsfA and RsfB. The *rsfA* and *rsfB* genes were expressed from an acetamide-inducible promoter on pMHRsfA and pMHRsfB, respectively. The expression level of *MSMEG_1777* was determined in the Δ*rsfA* mutant with pMHRsfA and the Δ*rsfB* mutant with pMHRsfB with increasing concentrations of acetamide in growth medium. As shown in Figures 7C,D, the expression level of *MSMEG_1777* in the *M. smegmatis* strains with either pMHRsfA or pMHRsfB were gradually increased with increasing concentrations of acetamide, and lower concentrations of acetamide were required for similar levels of *MSMEG_1777* induction in *M. smegmatis* with pMHRsfA compared to *M. smegmatis* with pMHRsfB. Western blotting analysis showed that the amount of expressed RsfB was proportional to the concentration of acetamide. We did not detect the expressed RsfA by Western blotting analysis in the concentration range of acetamide used in the experiment. Taken together, these results provide the strong evidence that both RsfA and RsfB function as anti-SigF antagonists in *M. smegmatis*.

We further examined whether the significantly reduced expression of *MSMEG_1777* in the Δ*rsfB* mutant is caused by the decreased expression of *sigF* or the increased expression of *rsbW1*. Using Western blotting analysis, the protein levels of expressed SigF and RsbW1 were determined in the WT, Δ*rsfA*, and Δ*rsfB* strains that were grown aerobically to an OD_600_ of 0.45–0.5 (Supplementary Figure S4). The Western blotting result showed that the protein levels of SigF and RsbW1 in the Δ*rsfA* and Δ*rsfB* mutant strains are not different from those in the WT strain, indicating that the cellular levels of SigF and RsbW1 in *M. smegmatis* are not decreased under SigF-non-activating conditions. This finding can be explained by the presence of a SigF-independent promoter immediately upstream of the *rsbW1*-*sigF* operon (Gebhard et al., 2008).

### The Functionality of RsfB Is Controlled Through Phosphorylation of Ser-63 by RsbW2

**FIGURE 8:**
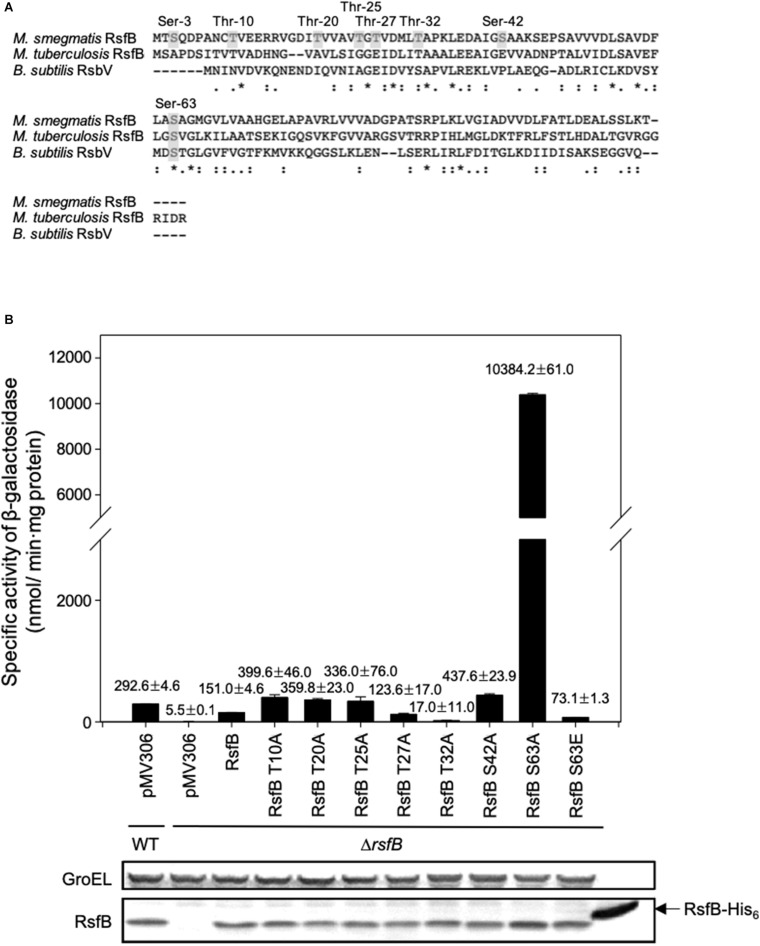
Identification of the amino acid residues that are responsible for the inactivation of RsfB by phosphorylation. **(A)** Multiple sequence alignment of the RsfB homologs of *M. smegmatis*, *M. tuberculosis*, and *B. subtilis* was generated using ClustalW. The asterisks and colons denote the conserved and conservatively substituted amino acid residues, respectively. The residues of *M. smegmatis* RsfB, which were identified to be phosphorylated by MSMEG_6129 *in vitro* (Bowman and Ghosh, 2014), are shown in the gray background. **(B)** Effects of T10A, T20A, T25A, T27A, S42A, S63A, and S63E mutations on the functionality of RsfB *in vivo*. The Δ*rsfB* strain harboring pNCII1777 was complemented with pMV306RsfB and its derivatives carrying the mutated *rsfB* gene (pMVRsfBT10A, pMVRsfBT20A, pMVRsfBT25A, pMVRsfBT27A, pMVRsfBT32A, pMVRsfBS42A, pMVRsfBS63A, pMVRsfBS63E). The complementation test was performed by determining the expression level of *MSMEG_1777* in the *M. smegmatis* strains. As controls, the *M. smegmatis* WT and Δ*rsfB* mutant strains with both pNCII1777 and the empty vector pMV306 were included in the experiment. The *M. smegmatis* strains were grown aerobically to an OD_600_ of 0.45–0.5 in 7H9-glucose medium. Cell-free crude extracts were used to measure β-galactosidase activity. All values are the means of the results from three biological replicates. The error bars indicate the standard deviations. Protein levels of the WT and mutant forms of RsfB expressed in the strains were detected by Western blotting analysis with RsfB polyclonal antibodies. As a loading control, GroEL was detected by a GroEL monoclonal antibody.

A previous study has reported that purified RsbW2 (MSMEG_6129) phosphorylates RsfB (MSMEG_6127) *in vitro* (Bowman and Ghosh, 2014). Using LC-tandem mass spectrometry, eight Ser/Thr residues (Ser-3, Thr-10, Thr-20, Thr-25, Thr-27, Thr-32, Ser-42, and Ser-63) in RsfB have been identified to be the phosphorylation sites by RsbW2 (Bowman and Ghosh, 2014). To specify the functionally important residue(s) among the identified phosphorylation sites in RsfB, we examined the functionality of the mutant forms of RsfB (T10A, T220A, T25A, T27A, T32A, S42A, S63A, and S63E) by determining *MSMEG_1777* expression in the Δ*rsfB* mutant expressing the mutant forms of RsfB. The Ser-3 was excluded from the experiment since the residue is not present in *Bacillus subtilis* RsbV (Figure 8A). The Δ*rsfB* mutant of *M. smegmatis* was complemented by introducing the pMV306 derivatives that carry the WT or mutant *rsfB* genes. The WT and Δ*rsfB* strains carrying the pMV306 empty vector were included in the experiment as positive and negative controls, respectively. As shown in Figure 8B, the S63A mutation led to a drastic increase in *MSMEG_1777* expression in *M. smegmatis*, whereas the T32A mutation resulted in abolishment of *MSMEG_1777* expression. The expression level of *MSMEG_1777* was shown to be decreased by about 50% in the *M. smegmatis* strain expressing the phosphomimetic S63E form of RsfB compared to the control *M. smegmatis* strain expressing WT RsfB. Western blotting analysis revealed that the WT and mutant forms of RsfB were expressed at similar levels in the *M. smegmatis* strains used in the experiment. These results suggest the followings: (i) unphosphorylated RsfB is the active form of RsfB as an anti-SigF antagonist, (ii) phosphorylation of RsfB on Ser-63 decreases the functionality of RsfB as in the RsfB homologs such as RsbV and SpoIIAA of *B. subtilis* (Bouillet et al., 2018), (iii) Thr-32 is important for anti-SigF antagonist activity of RsfB.

**FIGURE 9:**
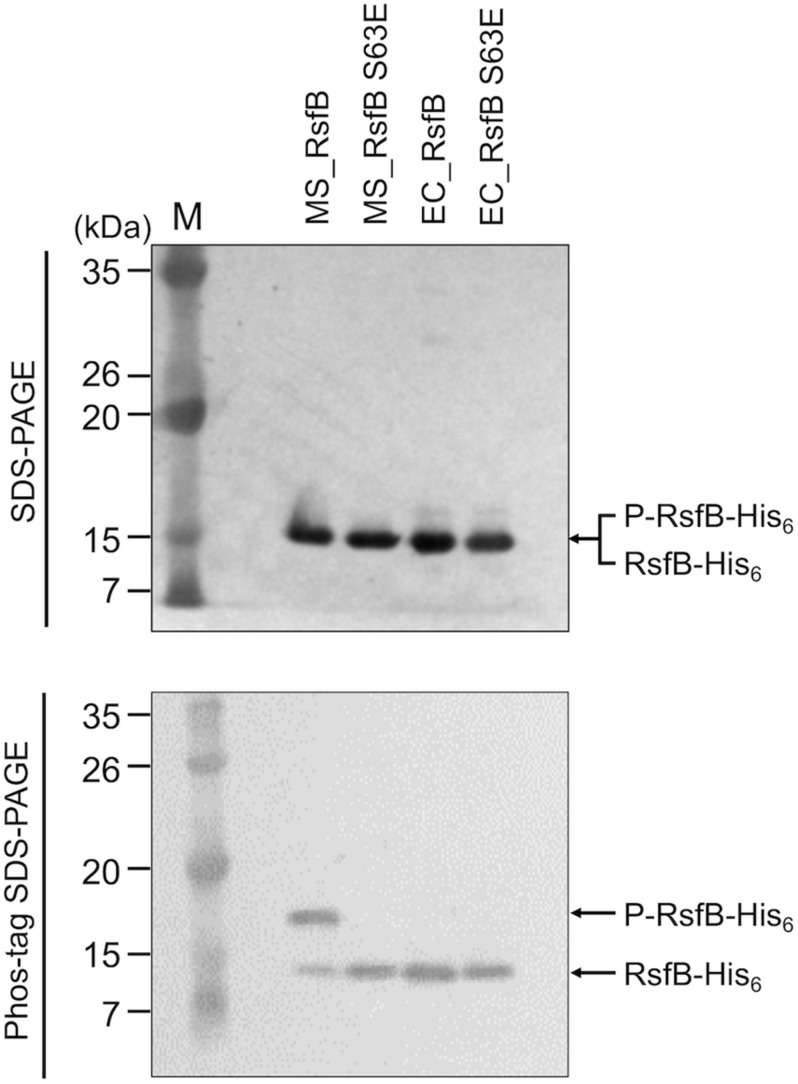
Phosphorylation state of the WT and S63E mutant forms of RsfB in *M. smegmatis* and *E. coli*. The WT and S63E mutant forms of His_6_-tagged RsfB were purified from *E. coli* and *M. smegmatis* that were aerobically grown to an OD_600_ of 0.4–0.5. 2 μg each of the WT and S63E mutant forms of RsfB were subjected to SDS-PAGE and Phos-tag SDS-PAGE, followed by Western blotting analysis with RsfB polyclonal antibodies. The bands representing unphosphorylated RsfB (RsfB-His_6_) and phosphorylated RsfB (P-RsfB-His_6_) are indicated by the arrows. MS_RsfB, RsfB purified from *M. smegmatis*. EC_RsfB, RsfB purified from *E. coli* M, molecular weight marker lanes.

To examine the phosphorylation state of RsfB in *M. smegmatis* grown under SigF-non-activating conditions and whether Ser-63 is the only residue that is phosphorylated, we expressed the WT and S63E mutant forms of His_6_-tagged RsfB in both *M. smegmatis* and *E. coli*, purified the proteins, and determined their phosphorylation state using Phos-tag SDS-PAGE analysis. As shown in Figure 9, most fractions of WT RsfB purified from *M. smegmatis* were found to be phosphorylated, while S63E RsfB purified from *M. smegmatis* was not phosphorylated at all. Both WT and S63E mutant forms of RsfB purified from *E. coli* were found to be unphosphorylated. The results indicate that Ser-63 in RsfB is the residue that is phosphorylated in *M. smegmatis*, and that *E. coli* does not have the protein kinase that can phosphorylate RsfB.

**FIGURE 10:**
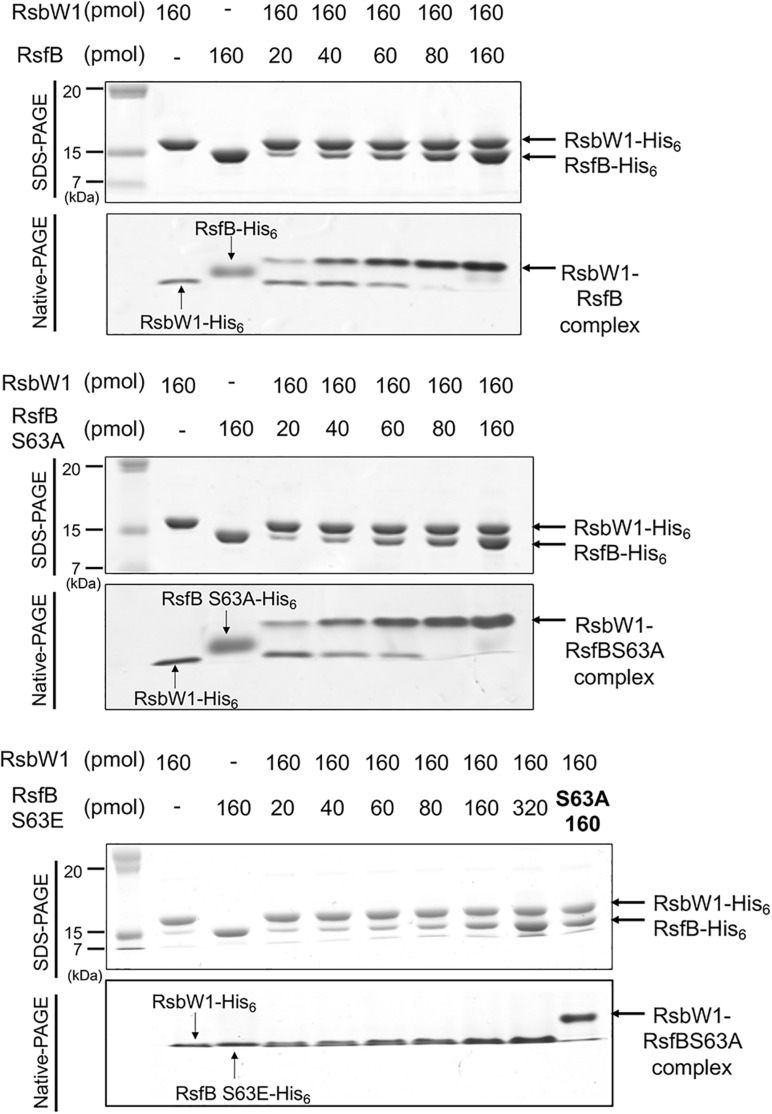
Determination of protein–protein interactions between RsbW1 and several forms of RsfB (WT, S63A, and S63E) by non-denaturing PAGE. 160 pmol of purified RsbW1 was mixed with increasing amounts of the purified WT and mutant forms (S63A and S63E) of RsfB in binding buffer [40 mM Tris–HCl (pH 8.0), 0.01 mM EDTA, 10 mM MgCl_2_, 20% (v/v) glycerol] and incubated for 30 min at 25°C. The mixtures were subjected to both SDS-PAGE (upper panel) and native PAGE (lower panel). The gels were stained with CBB. The bands representing RsbW1, RsfB (WT and mutant forms), and RsbW1-RsfB complex are indicated by the arrows.

We next examined the effect of Ser-63 phosphorylation on protein–protein interactions between RsfB and RsbW1. In place of phosphorylated RsfB, the phosphomimetic S63E mutant form of RsfB was employed for non-denaturing PAGE analysis. The WT and S63A RsfB proteins purified from *E. coli* were used as unphosphorylated RsfB. As shown in Figure 10, both WT RsfB and S63A RsfB interacted with purified RsbW1 and formed the retarded bands representing the RsfB-RsbW1 complex in non-denaturing PAGE. The intensity of the RsfB-RsbW1 complex bands was increased up to the ratio of RsbW1 to RsfB to be 1:1 with increasing amounts of WT RsfB and S63A RsfB. In contrast, the S63E mutant form of RsfB did not give rise to the RsfB-RsbW1 complex band even at high concentrations of S63E RsfB. The results suggest that phosphorylation of Ser-63 inactivates RsfB to render it unable to interact with RsbW1.

**FIGURE 11:**
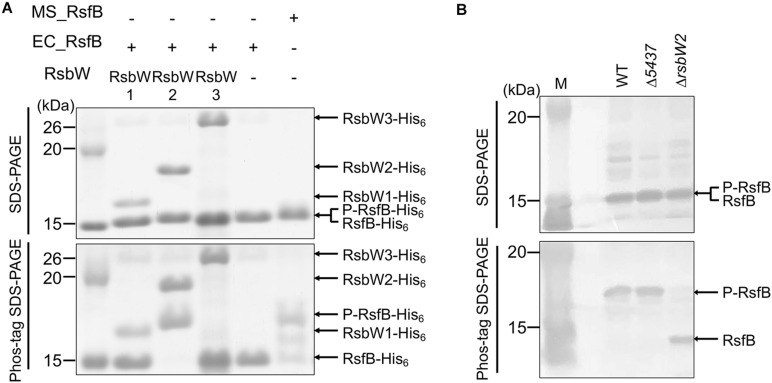
Determination of kinase activities of the RsbW homologs acting on RsfB. **(A)** RsfB purified from *E. coli* was mixed with partially purified RsbW1, purified RsbW2, or partially purified RsbW3 in reaction buffer [20 mM Tris–Cl (pH 7.5), 50 mM NaCl, 10 mM MgCl_2_, and 10 mM MnCl_2_]. The phosphorylation reactions were initiated by the addition of 100 μM ATP and performed for 30 min at 30°C. Subsequently, the reaction mixtures were subjected to both SDS-PAGE (upper panel) and Phos-tag SDS-PAGE (lower panel). The gels were stained with CBB. RsfB partially purified from *M. smegmatis* was used as the phosphorylated control of RsfB. The bands of the His_6_-tagged RsbW homologs, unphosphorylated RsfB (RsfB-His_6_), and phosphorylated RsfB (P-RsfB-His_6_) are indicated by the arrows. MS_RsfB, RsfB purified from *M. smegmatis*. EC_RsfB, RsfB purified from *E. coli*. **(B)** The phosphorylation state of the RsfB protein in the WT, Δ*5437*, and Δ*rsbW2* mutant strains of *M. smegmatis*. The strains were grown aerobically to an OD_600_ of 0.45–0.5 in 7H9-glucose medium. Cell-free crude extracts (60 μg) of the strains were separated simultaneously on both SDS-PAGE and Phos-tag SDS-PAGE, followed by Western blotting analysis with RsfB polyclonal antibodies. The bands representing unphosphorylated RsfB (RsfB) and phosphorylated RsfB (P-RsfB) are indicated by the arrows. M, molecular weight marker lanes.

Since the RsbW homologs of *Bacillus* SigB had been demonstrated to function as both anti-SigB and the protein kinase phosphorylating the anti-SigB antagonist (Benson and Haldenwang, 1993; Dufour and Haldenwang, 1994), we wondered whether in addition to RsbW2, RsbW1, and RsbW3 have the protein kinase activity phosphorylating RsfB. To examine this possibility, we performed *in vitro* kinase assay using purified RsbW homologs and RsfB. As shown in Figure 11A, only RsbW2 could phosphorylate unphosphorylated RsfB purified from *E. coli*, which is in good agreement with the fact that RsbW1 and RsbW3 are closely clustered with anti-sigma factors lacking the kinase activity, while RsbW2 is clustered with kinase-positive anti-sigma factors (Supplementary Figure S5). We also examined the phosphorylation state of RsfB in the WT and Δ*rsbW2* mutant strains grown under SigF-non-activating conditions using Phos-tag SDS-PAGE and Western blotting analysis (Figure 11B). The Δ*5437* mutant of *M. smegmatis* was included in the experiment, since it had been suggested that MSMEG_5437 is a Ser/Thr protein kinase that might modulate RsbW2 activity by phosphorylation (Bowman and Ghosh, 2014). Phos-tag SDS-PAGE showed that RsfB in the Δ*rsbW2* mutant was not phosphorylated in contrast to RsfB in the WT and Δ*5437* strains of *M. smegmatis*, indicating that RsbW2 is the only protein kinase that phosphorylates RsfB in *M. smegmatis*, and that RsbW2 is still active in the Δ*5437* mutant.

## Discussion

The genome of *M. smegmatis* contains three genes encoding RsbW homologs (RsbW1, RsbW2, and RsbW3). Among them, RsbW1 shows the highest degree of homology to UsfX that is a known anti-SigF in *M. tuberculosis*. Y2H and non-denaturing PAGE analysis revealed the interaction of RsbW1 with SigF, which is in good agreement with the previous result from bacterial two-hybrid assay (Singh et al., 2015). Overexpression of *rsbW1* in *M. smegmatis* led to a significant reduction in expression of *MSMEG_1777* that is under the control of SigF. Furthermore, disruption of the *rsbW1* gene by deletion resulted in an increase in yellow pigmentation of *M. smegmatis* colonies, which appears to be the result of increased isorenieratene biosynthesis. All of these results indicate that RsbW1 is a *bona fide* anti-SigF in *M. smegmatis*.

RsbW2 is most deviated among the RsbW homologs with regard to the reciprocal sequence homology (Supplementary Figure S5). In contrast to RsbW1, RsbW2 was shown not to interact with SigF in Y2H and non-denaturing PAGE analysis, implying that RsbW2 does not play a direct role as an anti-SigF. However, overexpression and inactivation of *rsbW2* gave rise to the same phenotype as those of *rsbW1* in terms of both *MSMEG_1777* expression and colony pigmentation. These results suggest that RsbW2 has an activity to decrease SigF functionality without direct binding to SigF. A clue about the anti-SigF activity of RsbW2 came from the kinase motifs (N, G1, and G2) that are conserved in RsbW2. Like *B. subtilis* RsbW that can inactivate the anti-SigB antagonist RsbV through phosphorylation (Dufour and Haldenwang, 1994), RsbW2 has the protein kinase activity that inhibits the functionality of RsfB by phosphorylation of Ser-63. RsfB was shown to exist in an unphosphorylated form in the Δ*rsbW2* mutant in contrast to the isogenic WT strain in which most fractions of RsfB exist in a phosphorylated form, which indicates that RsbW2 is the kinase that can phosphorylate RsfB in *M. smegmatis*. It is worth noting that RsbW of the SigB PSS in *Bacillus* species and *Streptomyces coelicolor* acts as both protein kinase for its cognate anti-SigB antagonist (RsbV) and anti-sigma factor (Benson and Haldenwang, 1993; Dufour and Haldenwang, 1994; Voelker et al., 1996; Lee et al., 2004; van Schaik and Abee, 2005), while RsbW1 and RsbW2 of *M. smegmatis* are specialized to function as anti-SigF and protein kinase, respectively.

Interestingly, both Δ*rsbW1* and Δ*rsbW2* mutants of *M. smegmatis* showed phenotypic instability in terms of yellow pigmentation. When first obtained, both the mutants exhibited yellow pigmentation on solid 7H9-glucose medium. However, when the mutant strains were passed through successive subcultures in solid and especially liquid growth media, the mutant strains lost yellow pigmentation and showed only basal levels of *MSMEG_1777* expression (data not shown). This observation implies that excessive expression of the SigF regulon is detrimental to *M. smegmatis*, leading to secondary mutations that mitigate the expression of the SigF regulon.

RsbW3 is closer to RsbW1 than RsbW2 in terms of sequence homology. In contrast to *rsbW1*, overexpression of *rsbW3* in *M. smegmatis* resulted in a significant increase in *MSMEG_1777* expression. Both the lack of protein–protein interactions between RsbW3 and SigF and the overexpression effect of *rsbW3* suggest the role of RsbW3 as an anti-SigF antagonist. Given both the quaternary structure (homodimers) of RsbW (SpoIIAB)-like anti-sigma factors (Campbell et al., 2002; Masuda et al., 2004) and the results demonstrating protein–protein interactions between RsbW1 and RsbW3 (Figure 6), we assume that RsbW3 likely inactivates RsbW1 by forming a heterodimer when overexpressed. The presence of a SigF–recognizing promoter (GTTT-N_17_-GGGTAA) upstream of *rsbW3* (Table S4) and abolishment of *rsbW3* expression by the inactivation of the *sigF* gene (Supplementary Figure S6) indicate that *rsbW3* belongs to the SigF regulon. Based on these findings, we suggest that RsbW3 serves as a booster for expression of the SigF regulon under SigF-activating conditions via the positive feedback loop.

So far, the roles of RsfA and RsfB as anti-SigF antagonists in mycobacteria have been predicted from both their protein interactions with anti-SigF (RsbW and UsfX) and the result from *in vitro* transcription analysis (Beaucher et al., 2002; Malik et al., 2008, 2009; Singh et al., 2015). Through both deletion and overexpression of *rsfA* and *rsfB*, we first demonstrated the physiological roles of RsfA and RsfB as anti-SigF antagonists in *M. smegmatis in vivo*. As judged by the RPKM values obtained from RNA sequencing analysis (Lee et al., 2018), the transcript level of *rsfB* was estimated to be ∼6-fold higher than that of *rsfA* in *M. smegmatis* grown aerobically to an OD_600_ of 0.4–0.5 in 7H9-glucose medium (Supplementary Figure S7). The difference in the expression levels of *rsfA* and *rsfB* might give a clue explaining the dominant role of RsfB as an anti-SigF antagonist. The observation that expression of *MSMEG_1777* was nearly abolished in the Δ*rsfB* mutant despite the presence of RsfA implies that the cellular level of active RsfA might not be sufficient to quarantine RsbW1 to such an extent as to induce the SigF regulon in the absence of RsfB. The result of *rsfA* and *rsfB* overexpression using an acetamide-inducible promoter clearly showed that when RsfA is sufficiently expressed, it acts as an anti-SigF antagonist more efficiently than RsfB. This observation is in good agreement with the previous report demonstrating that RsfA interacts more strongly with RsbW1 (UsfX) than RsfB (Singh et al., 2015). When the Δ*rsfA* mutant was complemented by introducing pMHRsfA (pMH201:*rsfA*), the strain did not grow at 0.01% acetamide in contrast to the Δ*rsfB* mutant carrying pMHRsfB (pMH201:*rsfB*) that grew, albeit slowly, even in the presence of 0.1% acetamide (data not shown). The inability of the Δ*rsfA* mutant with pMHRsfA to grow in the presence of 0.01% acetamide reinforces our assumption that expression of the SigF regulon in excess is toxic to *M. smegmatis*.

We found that Ser-63 is the amino acid residue of RsfB that is phosphorylated by RsbW2. It was also demonstrated that the S63A mutant form of RsfB interacts with RsbW1 with a similar affinity as the unphosphorylated form of WT RsfB, while the phosphomimetic (S63E) form of RsfB does not interact with RsbW1. These results confirm that the phosphorylation state of Ser-63 determines the functionality of RsfB as an anti-SigF antagonist. The importance of the corresponding serine residue in the functionality of anti-sigma factor antagonists has been reported for several RsfB homologs (RsfB of *M. tuberculosis*, RsbV and SpoIIAB of *B. subtilis*) (Diederich et al., 1994; Najafi et al., 1995; Yang et al., 1996; Beaucher et al., 2002).

The Δ*aa*_3_ mutant of *M. smegmatis* lacking the *aa*_3_ cytochrome *c* oxidase of the respiratory ETC has been reported to exhibit 53% of the oxygen consumption rate observed for the isogenic WT strain (Jeong et al., 2018), indicating that electron flow through the ETC is inhibited in the mutant by ∼50% relative to the WT strain. The finding that expression of the SigF regulon is significantly induced in the Δ*aa*_3_ mutant relative to the WT stain implies that the availability of free active SigF is increased in response to inhibition of the respiratory ETC. This observation is in good agreement with the suggestion that SigF makes direct contributions to transcriptomic remodeling in *M. smegmatis* under hypoxic growth conditions (Martini et al., 2019). The activation of SigF under respiration-inhibitory conditions might result from energy limitation as in the case of the SigB PSS in *Bacillus* species (Hecker and Volker, 2001; Marles-Wright and Lewis, 2007; de Been et al., 2011; Paget, 2015), or from other factors associated with ETC functions such as changes in the redox state of electron carriers, membrane potential, and proton motive force, etc. The inactivation of the *aa*_3_ cytochrome *c* oxidase in the background of the Δ*rsfB* mutant was shown not to lead to induction of *MSMEG_1777* expression. This result implies that RsfB mediates the induction of the SigF regulon under respiration-inhibitory conditions.

We demonstrated that the sole protein kinase that phosphorylates RsfB in *M. smegmatis* is RsbW2. RsbW2 has been suggested to be phosphorylated by a Ser/Thr protein kinase, MSMEG_5437 (Bowman and Ghosh, 2014), although the role of MSMEG_5437 in the SigF PSS remains elusive. In the vicinity of the *rsfB*-*rsbW2* operon occur the genes encoding a histidine kinase (MSMEG_6130) and a receiver domain-containing PP2C phosphatase (MSMEG_6131). The PP2C-family phosphatases are known to be responsible for dephosphorylation of the anti-SigB antagonist RsbV in *Bacillus* species and *S. coelicolor* (de Been et al., 2011). Indeed, our preliminary result showed that MSMEG_6131 could dephosphorylate the phosphorylated RsfB protein (data not shown). It is conceivable that the phosphorylation state of RsfB might be modulated by the combined control of the kinase activity of RsbW2 and the phosphatase activity of MSMEG_6131 that might be regulated by MSMEG_5437 Ser/The protein kinase and MSMEG_6130 histidine kinase, respectively. Further study is required to reveal the mechanism by which inhibition of the respiratory ETC leads to the activation of SigF in *M. smegmatis*.

## Data Availability Statement

The RNA sequencing data described in this study have been deposited in NCBI’s Gene Expression Omnibus and are accessible through the GEO Series accession number GSE155251.

## Author Contributions

J-IO, S-YS, and YO: conception or design of the study. YO, S-YS, H-JK, GH, and H-YK: acquisition of the data. YO, S-YS, JH, and J-IO: analysis or interpretation of the data and writing of the manuscript. All authors contributed to the article and approved the submitted version.

## Conflict of Interest

The authors declare that the research was conducted in the absence of any commercial or financial relationships that could be construed as a potential conflict of interest.
