# Supplementary material for: The Partner Switching System of the SigF Sigma Factor in Mycobacterium smegmatis and Induction of the SigF Regulon Under Respiration-Inhibitory Conditions
Source: Front Microbiol. 2020 Nov 11;11:588487. doi: 10.3389/fmicb.2020.588487 (PMC7693655; doi:10.3389/fmicb.2020.588487)
Supplement: Supplementary file 1 [file Data_Sheet_1.docx]

**SUPPLEMENTAL MATERIALS**

**The Partner Switching System of the SigF Sigma Factor in *Mycobacterium smegmatis* and Induction of the SigF Regulon under Respiration-Inhibitory Conditions**

Yuna Oh,*^1 ,†^* Su-Yeon Song,*^1, †^* Hye-Jun Kim,*^1^* Gil Han, *^1^* Jihwan Hwang,*^1^* Ho-Young Kang,*^1^* and Jeong-Il Oh*^1*^*

*^1^Department of Integrated Biological Science, Pusan National University, 46241 Busan, Korea*

*^†^ These authors have contributed equally to this work*

***Correspondence:**

Jeong-Il Oh

joh@pusan.ac.kr

Running title: SigF Partner Switching System in *Mycobacterium smegmatis*

**Keywords: *aa_3_* cytochrome *c* oxidase, anti-sigma factor, anti-anti-sigma factor, electron transport chain, *Mycobacterium*, partner switching system, protein kinase, regulation of gene expression, SigF**

**MATERIALS AND METHODS**

**Construction of mutant strains of *M. smegmatis*** Deletion mutants of *M. smegmatis* were constructed by allelic exchange mutagenesis using the suicide vector pKOTs containing a temperature-sensitive replication origin as described previously (Jeong et al., 2013). In brief, the temperature-sensitive suicide plasmid was introduced into the WT or Δ*aa_3_* mutant strains of *M. smegmatis* by electroporation. Transformants were selected at 30°C (replication-permissive temperature) on 7H9-glucose agar plates containing hygromycin, and the selected transformants were grown in 7H9-glucose liquid medium supplemented with hygromycin for 3 - 5 days at 30°C. Heterogenotes of *M. smegmatis*, which were generated by a single recombination event, were selected for their hygromycin resistance on 7H9-glucose agar plates at 42°C (replication-nonpermissive temperature). The selected heterogenotes were grown on 7H9-glucose medium without antibiotics for 3 - 5 days at 37°C. Isogenic homogenotes were obtained from the heterogenotes after a second recombination by selecting them for sucrose resistance on 7H9-glucose agar plates containing 10% (w/v) sucrose at 37°C. The allelic exchange was verified by PCR with isolated genomic DNA (Fig. S1).

**(i) Δ*rsfA* and** Δ***aa_3_***Δ***rsfA* mutants.** To construct the Δ*rsfA* and Δ*aa_3_*Δ*rsfA* mutants of *M. smegmatis*, the allelic exchange using pKOTsΔrsfA was performed in the WT and Δ*aa_3_* strains of *M. smegmatis*, respectively.

**(ii) Δ*rsfB* and** Δ***aa_3_***Δ***rsfB* mutants.** To construct the Δ*rsfB* and Δ*aa_3_*Δ*rsfB* mutants of *M. smegmatis*, the allelic exchange using pKOTsΔrsfB was performed in the WT and Δ*aa_3_* strains of *M. smegmatis*, respectively.

**(iii)** Δ***aa_3_***Δ***sigF* mutant.** To construct the Δ*aa_3_*Δ*sigF* mutant of *M. smegmatis*, the allelic exchange using pKOTsΔsigF was performed in the Δ*aa_3_* strain of *M. smegmatis*.

**(iv) Δ*rsbW1*, Δ*rsbW2*, Δ*rsbW3*, and Δ*5437* mutants.** To construct the Δ*rsbW1*, Δ*rsbW2*, Δ*rsbW3*, and Δ*5437* mutant strains of *M. smegmatis*, the allelic exchange using pKOTsΔrsbW1, pKOTsΔrsbW1, pKOTsΔrsbW1, and pKOTsΔ5437 was performed in the WT strain of *M. smegmatis*, respectively.

**Construction of plasmids**

**(i) The temperature-sensitive suicide plasmids for the construction of mutant strains of *M. smegmatis.*** For the construction of pKOTsΔrsfA, two rounds of recombination PCR were conducted. Using the chromosomal DNA of *M. smegmatis* mc^2^155 as a template, two primary PCR reactions were performed with the primers F_rsfA_mut and R_rsfA_rec, as well as with the primers F_rsfA_rec and R_rsfA_mut to generate two 40-bp overlapping DNA fragments (406 and 390 bp, respectively). Both PCR products contain the same 182-bp deletion within *rsfA* in the overlapping region. In the secondary PCR, a 756-bp DNA fragment with in-frame deletion of *rsfA* was obtained using both the primary PCR products as templates and the F_rsfA_mut and R_rsfA_mut primers. The secondary PCR product was restricted with NotI and HindIII, and cloned into pKOTs digested with the same enzymes, yielding pKOTsΔrsfA.

For the construction of pKOTsΔrsfB, two rounds of recombination PCR were conducted. Using the chromosomal DNA of *M. smegmatis* mc^2^155 as a template, two primary PCR reactions were performed with the primers F_rsfB_mut and R_rsfB_rec, as well as with the primers F_rsfB_rec and R_rsfB_mut to generate two 40-bp overlapping DNA fragments (388 and 393 bp, respectively). Both PCR products contain the same 208-bp deletion within *rsfB* in the overlapping region. In the secondary PCR, a 741-bp DNA fragment with in-frame deletion of *rsfB* was obtained using both the primary PCR products as templates and the F_rsfB_mut and R_rsfB_mut primers. The secondary PCR product was restricted with NotI and HindIII, and cloned into pKOTs digested with the same enzymes, yielding pKOTs∆rsfB.

For the construction of pKOTsΔrsbW1, two rounds of recombination PCR were conducted. Using the chromosomal DNA of *M. smegmatis* mc^2^155 as a template, two primary PCR reactions were performed with the primers F_rsbW1_mut and R_rsbW1_rec, as well as with the primers F_rsbW1_rec and R_rsbW1_mut to generate two 39-bp overlapping DNA fragments (345 and 804 bp, respectively). Both PCR products contain the same 291-bp deletion within *rsbW1* in the overlapping region. In the secondary PCR, a 1110-bp DNA fragment with in-frame deletion of *rsbW1* was obtained using both the primary PCR products as templates and the F_rsbW1_mut and R_rsbW1_mut primers. The secondary PCR product was restricted with NotI and HindIII, and cloned into pKOTs digested with the same enzymes, yielding pKOTsΔrsbW1.

For the construction of pKOTsΔrsbW2, two rounds of recombination PCR were conducted. Using the chromosomal DNA of *M. smegmatis* mc^2^155 as a template, two primary PCR reactions were performed with the primers F_rsbW2_mut and R_ rsbW2_rec, as well as with the primers F_rsbW2_rec and R_rsbW2_mut to generate two 37-bp overlapping DNA fragments (366 and 375 bp, respectively). Both PCR products contained the same 373-bp deletion within *rsbW2* in the overlapping region. In the secondary PCR, a 761-bp DNA fragment with in-frame deletion of *rsbW2* was obtained using both the primary PCR products as templates and the F_rsbW2_mut and R_rsbW2_mut primers. The secondary PCR product was restricted with NotI and HindIII, and cloned into pKOTs, yielding pKOTsΔrsbW2.

For the construction of pKOTsΔrsbW3, two rounds of recombination PCR were conducted. Using the chromosomal DNA of *M. smegmatis* mc^2^155 as a template, two primary PCR reactions were performed with the primers F_rsbW3_mut and R_rsbW3_rec, as well as with the primers F_rsbW3_rec and R_rsbW3_mut to generate two 36-bp overlapping DNA fragments (457 and 420 bp, respectively). Both PCR products contained the same 435-bp deletion within *rsbW3* in the overlapping region. In the secondary PCR, a 841-bp DNA fragment with in-frame deletion of *rsbW3* was obtained using both the primary PCR products as templates and the F_rsbW3_mut and R_rsbW3_mut primers. The secondary PCR product was restricted with NotI and HindIII, and cloned into pKOTs, yielding pKOTsΔrsbW3.

For the construction of pKOTsΔ5437, two rounds of recombination PCR were conducted. Using the chromosomal DNA of *M. smegmatis* mc^2^155 as a template, two primary PCR reactions were performed with the primers, F_5437_mut and R_5437_rec, as well as with the primers, F_5437_rec and R_5437_mut, to generate two 37-bp overlapping DNA fragments (401 and 472 bp, respectively). Both PCR products contained the same 762-bp deletion within *MSMEG_5437* in the overlapping region. In the secondary PCR, a 836-bp DNA fragment with in-frame deletion of *5437* was obtained using both the primary PCR products as templates and the F_5437_mut and R_5437_mut primers. The secondary PCR product was restricted with HindIII and NotI, and cloned into pKOTs, yielding pKOTs∆5437.

**(ii) pNCII.** Using the *lacZ* transcriptional fusion plasmid pNC as a template, inverse PCR was performed with the primers Left_pNC_BamHI and Right_pNC_BamHI to remove the ribosome binding site (RBS) of the *lacZ* gene on pNC, resulting in a linear pNC fragment without RBS of the *lacZ* gene. The PCR product was restricted with BamHI and self-ligated, yielding the *lacZ* translational fusion plasmid pNCII.

**(iii) pNCII1777.** pNCII1777 is a *MSMEG_1777*::*lacZ* translational fusion plasmid. For the construction of pNCII1777, a 464-bp DNA fragment comprising the 5’ portion (54 bp) of *MSMEG_1777* and 392-bp DNA sequence upstream of *MSMEG_1777* was amplified with F_1777lacZ_XbaI and R_1777lacZ_BamHI, using the chromosomal DNA of *M. smegmatis* mc^2^155 as a template and *Pfu* DNA polymerase. The PCR product was restricted with XbaI and BamHI and cloned into pNCII, yielding pNCII1777.

**(iv) pBSIIrsfB.** For the construction of pBSIIrsfB, a 949-bp DNA fragment containing the *rsfB* gene *(MSMEG_6127)* was amplified by PCR with the primers F_rsfB_com_BamHI and R_rsfB_mut, using the chromosomal DNA of *M. smegmatis* mc^2^155 as a template. The PCR product was restricted with BamHI and HindIII and cloned into pBSII, resulting in pBSIIrsfB.

**(v) pMVRsfB.** pMVRsfB was constructed for complementation of the Δ*rsfB* mutant strain. pMVRsfB was constructed by cloning of a 953-bp XbaI and HindIII fragment form pBSIIrsfB into pMV306.

**(vi) pMVRsfBT10A, pMVRsfBT20A, pMVRsfBT25A, pMVRsfBT27A, pMVRsfBT32A, pMVRsfBS42A, pMVRsfBS63A, and pMVRsfBS63E.** To introduce point mutations (T10A, T20A, T25A, T27A, T32A, S42A, S63A and S63E) into *rsfB*, PCR-based site-directed mutagenesis was performed using pMVRsfB as a template and the primers listed in Table S2.

**(vii) pT7-7sigF, pT7-7rsbW1, pT7-7rsbW2, pT7-7rsbW3, pT7-7rsfA, and pT7-7rsfB.** A 771-bp DNA fragment encompassing the *sigF* gene and six His codons immediately before its stop codon was amplified by PCR with the primers F_sigFover and R_sigFover, using the chromosomal DNA of *M. smegmatis* mc^2^155 as a template and *Pfu* DNA polymerase. The PCR product was restricted with NdeI and BamHI and cloned into pT7-7, yielding pT7-7sigF.

A 450-bp DNA fragment encompassing the *rsbW1* gene and six His codons immediately before its stop codon was amplified by PCR with the primers F_rsbW1over and R_rsbW1over, using the chromosomal DNA of *M. smegmatis* mc^2^155 as a template and *Pfu* DNA polymerase. The PCR product was restricted with NdeI and PstI and cloned into pT7-7, yielding pT7-7rsbW1.

A 482-bp DNA fragment encompassing the *rsbW2* gene and six His codons immediately before its stop codon was amplified by PCR with the primers F_rsbW2over and R_rsbW2over, using the chromosomal DNA of *M. smegmatis* mc^2^155 as a template and *Pfu* DNA polymerase. The PCR product was restricted with NdeI and HindIII and cloned into pT7-7, yielding pT7-7rsbW2.

A 620-bp DNA fragment encompassing the *rsbW3* gene and six His codons immediately before its stop codon was amplified by PCR with the primers F_rsbW3over and R_rsbW3over, using the chromosomal DNA of *M. smegmatis* mc^2^155 as a template and *Pfu* DNA polymerase. The PCR product was restricted with NdeI and BamHI and cloned into pT7-7, yielding pT7-7rsbW3.

A 464-bp DNA fragment encompassing the *rsfA* gene and six His codons immediately before its stop codon was amplified by PCR with the primers F_rsfAover and R_rsfAover, using the chromosomal DNA of *M. smegmatis* mc^2^155 as a template and *Pfu* DNA polymerase. The PCR product was restricted with NdeI and HindIII and cloned into pT7-7, yielding pT7-7rsfA.

A 395-bp DNA fragment encompassing the *rsfB* gene and six His codons immediately before its stop codon was amplified by PCR with the primers F_rsfBover and R_rsfBover, using the chromosomal DNA of *M. smegmatis* mc^2^155 as a template and *Pfu* DNA polymerase. The PCR product was restricted with NdeI and HindIII and cloned into pT7-7, yielding pT7-7rsfB.

**(viii)** **pMHRsbW1, pMHRsbW2, pMHRsbW3, pMHRsfA, and pMHRsfB**. The DNA fragments resulting from pT7-7rsbW1, pT7-7rsbW2, pT7-7rsbW3, pT7-7rsfA, and pT7-7rsfB by restriction of NdeI and ClaI were cloned into the pMH201 integration vector with an acetamide-inducible promoter, resulting in pMHRsbW1, pMHRsbW2, pMHRsbW3, pMHRsfA, and pMHRsfB, respectively.

**(ix)** **pT7-7rsfBS63A, pT7-7rsfB63E, and pMHRsfBS63E.** To introduce point mutations (S63A and S63E) into RsfB, PCR-based site-directed mutagenesis was performed using pT7-7rsfB as a template and the primers listed in Table S2. In order to construct pMHRsfBS63E, pT7-7rsfBS63E was digested with NdeI and ClaI, and the resulting DNA fragment with the *rsfB* gene was cloned into pMH201, yielding pMHRsfBS63E.

**(x) pGBKSigF and pGBKRsbW3.** A 770-bp DNA fragment encompassing the *sigF* gene was amplified by PCR with the primers F_sigF_NdeI and R_sigF_BamHI, using the chromosomal DNA of *M. smegmatis* mc^2^155 as a template and *Pfu* DNA polymerase. The PCR product was restricted with NdeI and BamHI and cloned into pGBKT7, yielding pGBKSigF.

A 602-bp DNA fragment containing the *rsbW3* gene was amplified by PCR with the primers F_rsbW3over and R_rsbW3_BamHI, using the chromosomal DNA of *M. smegmatis* mc^2^155 as a template and *Pfu* DNA polymerase. The PCR product was restricted with NdeI and BamHI and cloned into pGBKT7, yielding pGBKRsbW3.

**(xi) pPLRsbW1, pPLRsbW2, and pPLRsbW3.** A 437-bp DNA fragment containing the *rsbW1* gene was amplified by PCR with the primers F_rsbW1_EcoRI and R_rsbW1_XhoI, using the chromosomal DNA of *M. smegmatis* mc^2^155 as a template and *Pfu* DNA polymerase. The PCR product was restricted with EcoRI and XhoI and cloned into pGADT7linker, resulting in pPLRsbW1.

A 469-bp DNA fragment encompassing the *rsbW2* gene was amplified by PCR with the primers F_rsbW2_BamHI and R_rsbW2_XhoI, using the chromosomal DNA of *M. smegmatis* mc^2^155 as a template and *Pfu* DNA polymerase. The PCR product was restricted with BamHI and XhoI and cloned into pGADT7linker, yielding pPLRsbW2.

A 607-bp DNA fragment encompassing the *rsbW3* gene (*MSMEG_1787*) was amplified by PCR with the primers F_rsbW3_BamHI and R_rsbW3_XhoI, using the chromosomal DNA of *M. smegmatis* mc^2^155 as a template and *Pfu* DNA polymerase. The PCR product was restricted with BamHI and XhoI and cloned into pGADT7linker, yielding pPLRsbW3.


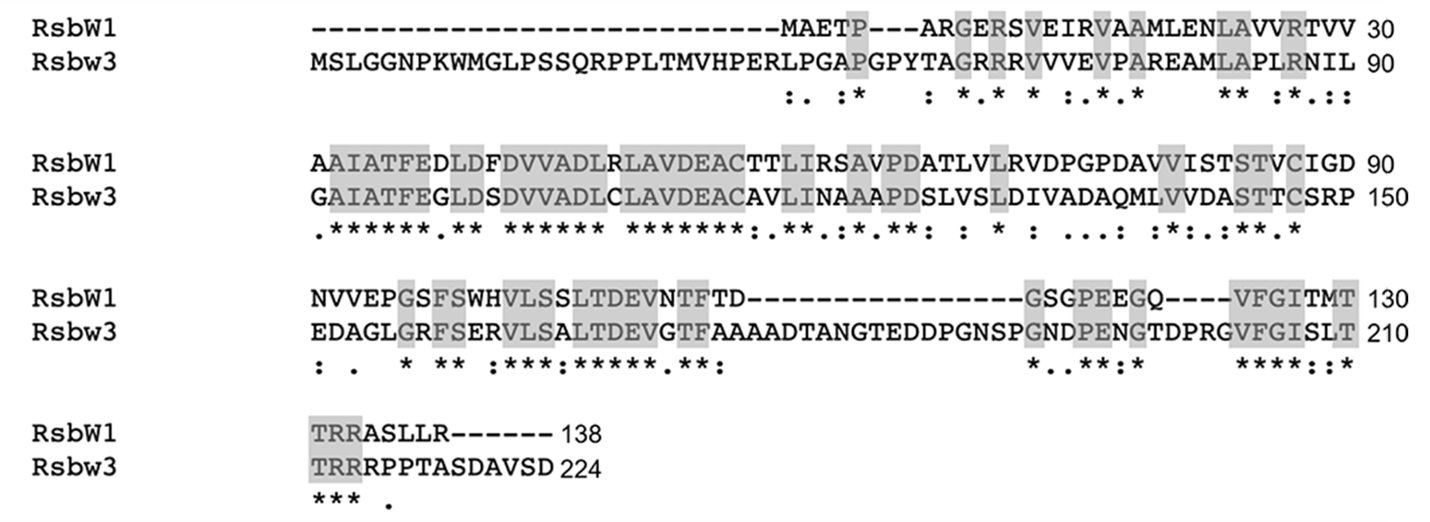


**FIGURE S1. Sequence alignment of RsbW1 and RsbW3.** Alignment of RsbW1 (MSMEG_1803) and RsbW3 (MSMEG_1787) was generated using ClustalW. The identical amino acids are indicated by asterisks and the gray background. The conserved and semiconserved substitutions are indicated by colons and dots, respectively.
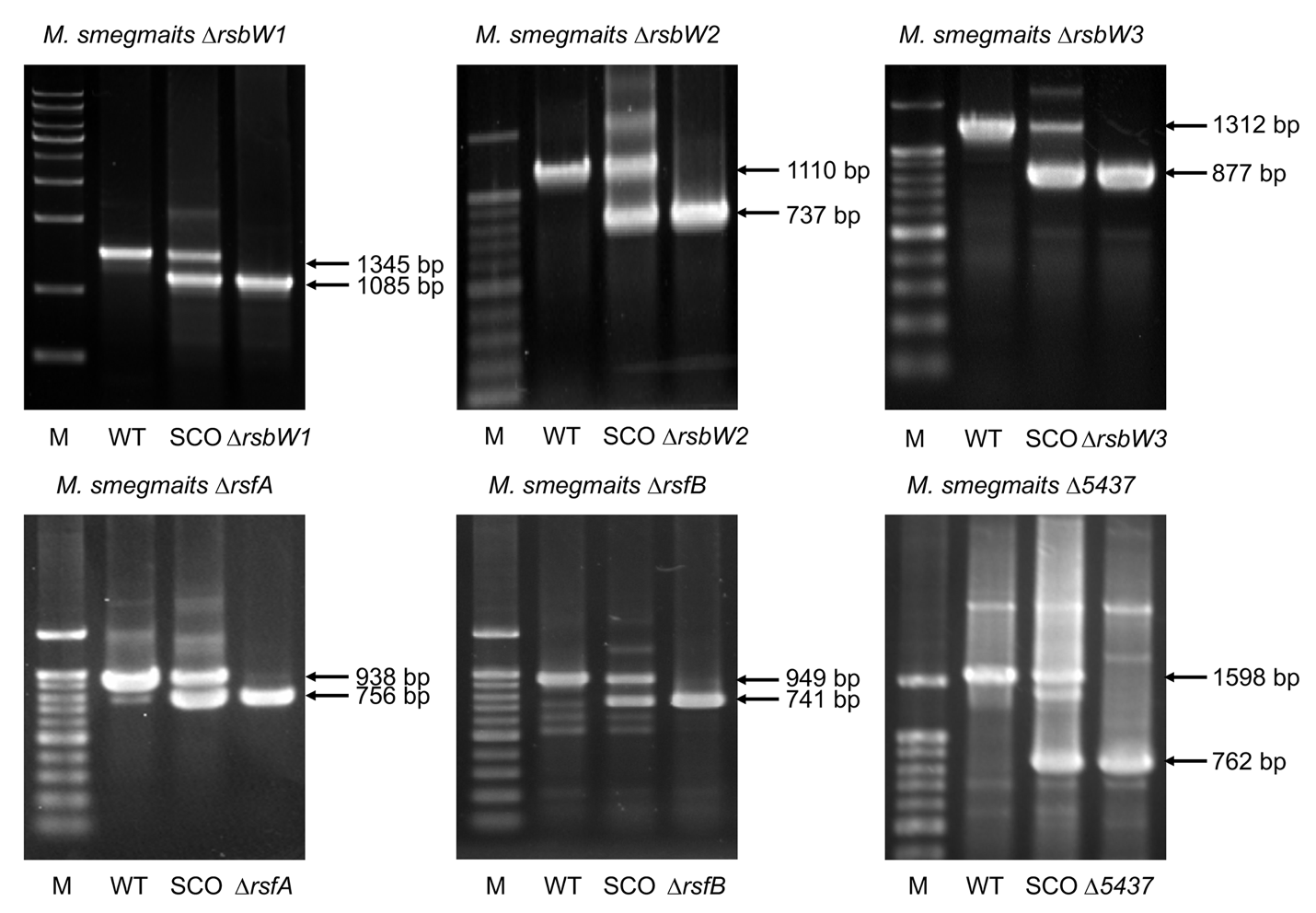


**FIGURE S2. Validation of the *rsbW1, rsbW2, rsbW3, rsfA,* *rsfB,* and *MSMEG_5437* deletion mutants of *M. smegmatis* by PCR.** The PCR reactions were performed with gene-specific primers using the chromosomal DNA from the wild-type (WT), single crossover (SCO), and deletion mutant (Δ*rsbW1*, Δ*rsbW2*, Δ*rsbW3*, Δ*rsfA*, Δ*rsfB,* and Δ*5437*) strains. The expected sizes of the PCR products are marked with the arrows. The primer sets employed for confirmation of deletions are listed in Table S2. M, DNA size marker lanes**.**


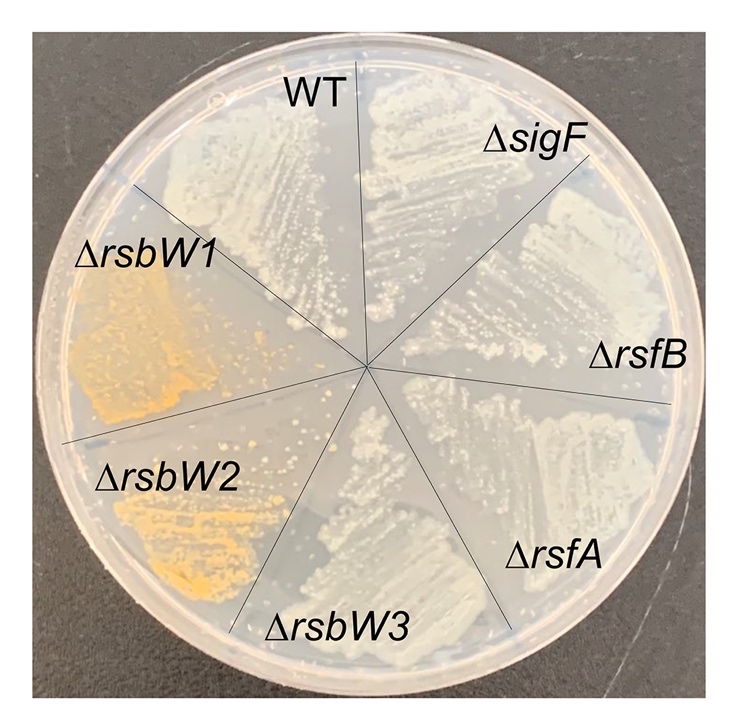


**FIGURE S3. Colony pigmentation of the *M. smegmatis* mutants related to the SigF PSS.** The WT and Δ*sigF*, Δ*rsbW1*, Δ*rsbW2*, Δ*rsbW3*, Δ*rsfA*, and Δ*rsfB* mutant strains of *M. smegmatis* were streaked on solid 7H9-glucose medium and grown at 37°C.

**
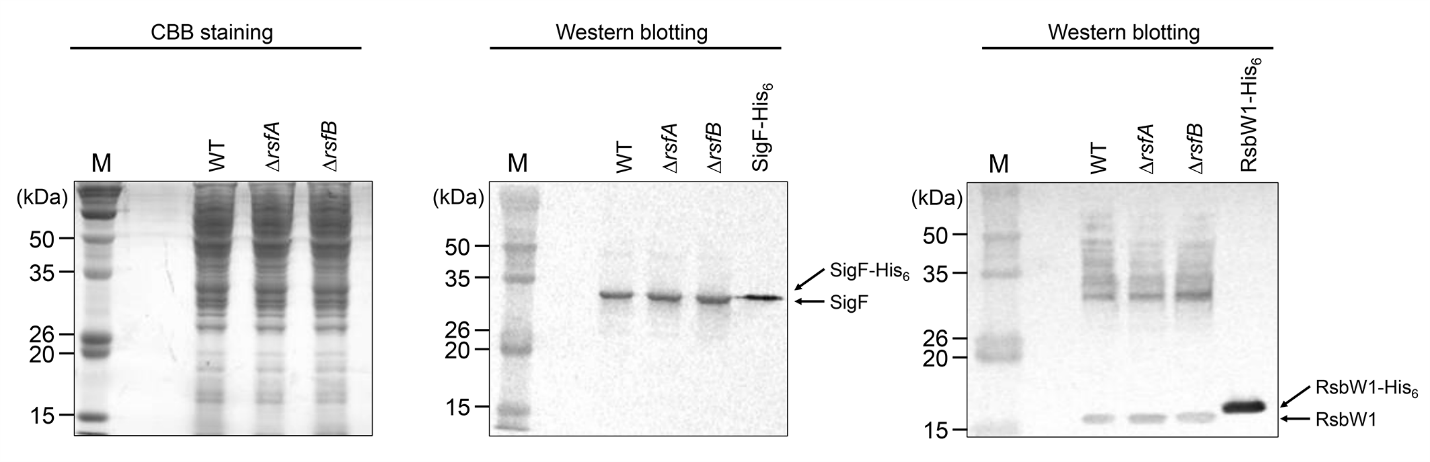
**

**FIGURE S4. Protein levels of expressed SigF and RsbW1 in the WT, Δ*rsfA*, and Δ*rsfB* mutant strains of *M. smegmatis*.** The WT and mutant strains of *M. smegmatis* were grown aerobically to an OD_600_ of 0.45 - 0.5 in 7H9-glucose medium. Cell-free crude extracts (15 μg) of the strains and purified His_6_-tagged proteins (0.1 μg) were separated on SDS-PAGE, followed by Western blotting analysis with SigF and RsbW1 polyclonal antibodies. The CBB-stained gel is included as the loading control of the crude extracts. The bands representing SigF and RsbW1 are indicated by the arrows. M, molecular weight marker lanes.


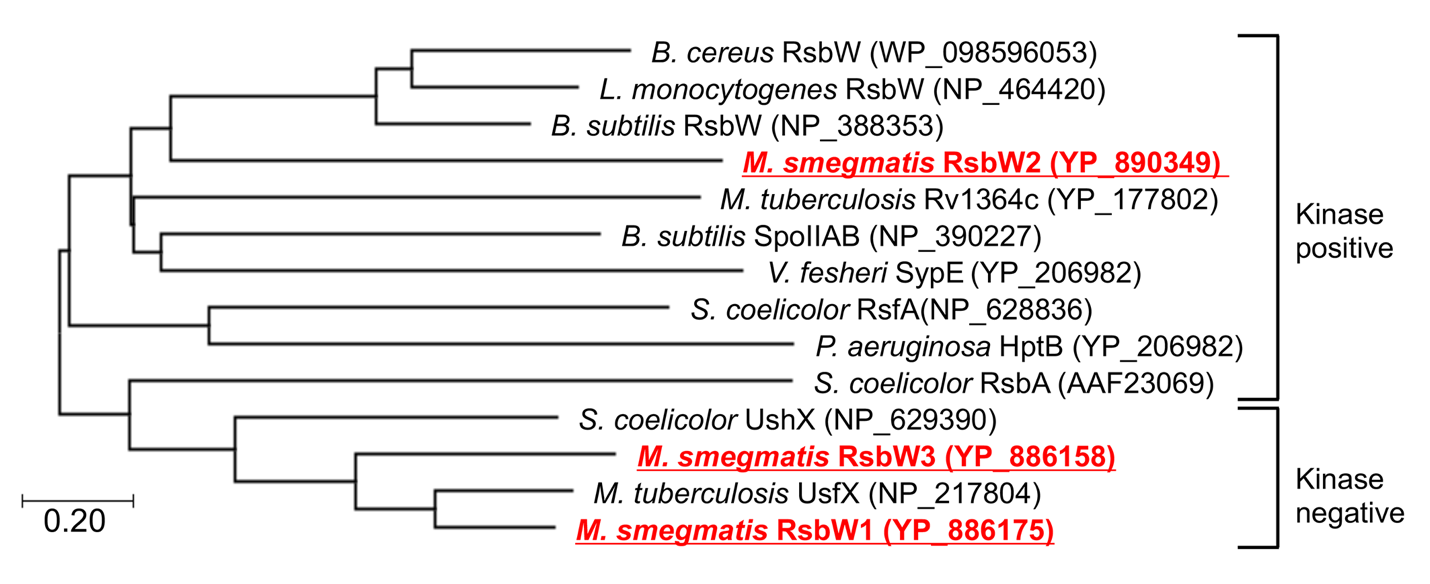
 **FIGURE S5. The neighbor-joining phylogenetic tree of RsbW-related anti-sigma factors from various bacterial species.** Phylogenetic analysis was performed using the MEGA software (v10.1.1). The marked distance bar indicates 0.2 amino acid substitutions per site. The genebank accession numbers for the proteins are given in parentheses after their names. Three RsbW homologs in *M. smegmatis* are highlighted with red fonts and underlines.

**_
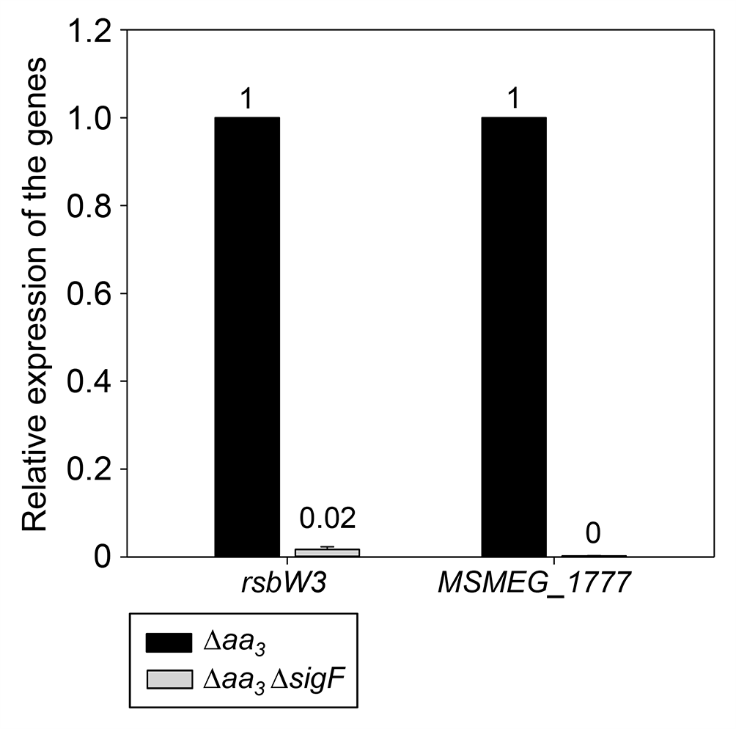
_**

**FIGURE S6. The relative expression levels of the *rsbW3* and *MSMEG_1777* genes in the Δ*aa_3_*Δ*sigF* strain relative to the Δ*aa_3_* strain.** Total RNA was obtained from the Δ*aa_3_* and Δ*aa_3_*Δ*sigF* strains grown aerobically to an OD_600_ of 0.45 - 0.5 in 7H9-glucose medium. The expression level of *rsbW3* and *MSMEG_1777* determined by qRT-PCR was normalized to that of *sigA* (the constitutively expressed gene encoding the principal sigma factor). The expression level of each gene in Δ*aa_3_* mutant strain is set at 1, and the relative value is expressed for that in Δ*aa_3_*Δ*sigF* mutant strain. All values given are the means of the results from three biological replicates.

**
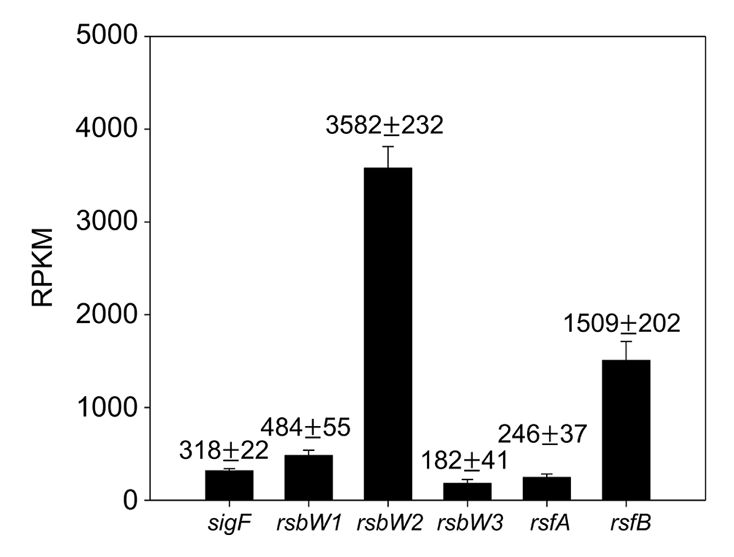
**

**FIGURE S7. The transcript levels of *sigF*, *rsbW1*, *rsbW2*, *rsbW3*, *rsfA*, and *rsfB* in the WT strain of *M. smegmatis*.** The transcript levels of the *sigF*, *rsbW1*, *rsbW2*, *rsbW3*, *rsfA*, and *rsfB* genes were extrapolated from their RPKM values obtained from RNA sequencing analysis on the WT strain of *M. smegmatis* that was aerobically grown to an OD_600_ of 0.45 - 0.6 in 7H9-glucose medium (Lee et al., 2018). The RPKM values are the means of the results from three biological replicates. The error bars indicate the standard deviations.

**TABLE S1.** Strains and plasmids used in this study

| **Strain/plasmid** | **Relevant phenotype/genotype^a^** | **Reference** |
| --- | --- | --- |
| Strains |  |  |
| *E. coli* DH5α | φ80dl*acZ*ΔM15 Δ*lacU169* *recA1* *endA1* *hsdR17* *supE44 thi1 gyrA96 relA1* | Jessee, 1986 |
| *E. coli* BL21 (DE3) | F^-^ *ompT hsd*S*_B_* (r_B_^-^ m_B_^-^) *dcm gal* λ (DE3) | Promega |
| *E. coli* Rosetta-gami™ 2 (DE3) | Δ*(ara-leu)7697* Δ*lacX74* Δ*phoA* PvuII *phoR araD139 ahpC galE galK rpsL* (DE3) *F′[lac+ lacIq pro] gor522::Tn10 trxB* pLysSRARE2 (Cam^r^, Str^r^, Tet^r^) | Novagen |
| *S. cerevisiae* AH109 | *MATa, trp1-901, leu2-3, 112, ura3-52, his3-200, gal4Δ, gal80Δ, LYS2::GAL1_UAS_- GAL1_TATA_-HIS3, GAL2_UAS_-GAL2_TATA_-ADE2, URA3::MEL1_UAS_-MEL1_TATA_-lacZ* | James et al., 1996 |
| *M. smegmatis* Δ*rsbW3* | *MSMEG_1787* (*rsbW3)* deletion mutant derived from *M. smegmatis* mc^2^155 | This study |
| *M. smegmatis* Δ*rsfA* | *MSMEG_1786* (*rsfA*) deletion mutant derived from *M*. *smegmatis* mc^2^155 | This study |
| *M. smegmatis* Δ*rsfB* | *MSMEG_6127* (*rsfB*) deletion mutant derived from *M*. *smegmatis* mc^2^155 | This study |
| *M. smegmatis* Δ*5437* | *MSMEG_5437* deletion mutant derived from *M*. *smegmatis* mc^2^155 | This study |
| *M. smegmatis* Δ*f1f2f3* | *MSMEG_6383 (furA1), MSMEG_3460 (furA2)*, and *MSMEG_6253 (furA3)* triple-deletion mutant derived from *M. smegmatis* mc^2^155 | Lee et al., 2018 |
| *M. smegmatis* Δ*aa*_3_ | *MSMEG_4268* (*ctaC*) deletion mutant derived from *M*. *smegmatis* mc^2^155 | Jeong et al., 2018 |
| *M. smegmatis* Δ*aa*_3_Δ*rsfA* | *MSMEG_4268* (*ctaC*) and *MSMEG_1786* (*rsfA*) double-deletion mutant derived from *M*. *smegmatis* mc^2^155 | This study |
| *M. smegmatis* Δ*aa*_3_Δ*rsfB* | *MSMEG_4268* (*ctaC*) and *MSMEG_6127* (*rsfB*) double-deletion mutant derived from *M*. *smegmatis* mc^2^155 | This study |
| *M. smegmatis* Δ*aa*_3_Δ*sigF* | *MSMEG_4268* (*ctaC*) and *MSMEG_1804* (*sigF*) double-deletion mutant derived from *M*. *smegmatis* mc^2^155 | This study |
| Plasmids |  |  |
| pBluescript II KS + | Amp^r^; *lacPOZ’* | Stratagene |
| pNC | Hyg^r^; promoterless *lacZ* | Oh et al., 2010 |
| pNCII | Hyg^r^; a derivative of pNC, the ribosome-binding site of *lacZ* is removed (translational fusion) | This study |
| pT7-7 | Amp^r^; T7 promoter, ribosome binding site, and translation start codon overlapping with NdeI site | Tabor and Richardson, 1985 |
| pMH201 | Km^r^; acetamide-inducible promoter, derivative of pMV306 | Kang et al., 2005 |
| pKOTs | Hyg^r^; pKO-based vector constructed by inserting HindIII-KpnI fragment containing pAL500Ts and the pUC ori derived from pDE | Jeong et al., 2013 |
| pGBKT7 | Km^r^; *TRP1*, GAL4 DNA-binding domain | Louvet et al., 1997 |
| pGADT7linker | Amp^r^; *LEU2*, GAL4 activation domain | Lee et al., 2012 |
| pMV306 | Km^r^; integration vector containing *int* and *attP* site of mycobacteriophage L5 for integration into the mycobacterial genome | Stover et al., 1991; Brown et al., 2007 |
| pBSIIrsfB | pBluescript II KS+::1.041-kb BamHI-HindIII fragment containing *rsfB* | This study |
| pNCII1777 | pNCII::0.456-kb XbaI-BamHI fragment containing the *MSMEG_1777* promoter region | This study |
| pT7-7sigF | pT7-7::0.437-kb NdeI-BamHI fragment containing *sigF* (*MSMEG_1804*) with six His codons before its stop codons | This study |
| pT7-7rsbW1 | pT7-7::0.437-kb NdeI-PstI fragment containing *rsbW1* (*MSMEG_1803*) with six His codons before its stop codons | This study |
| pT7-7rsbW2 | pT7-7::0.471-kb NdeI-HindIII fragment containing *rsbW2* (*MSMEG_6129*) with six His codons before its stop codons | This study |
| pT7-7rsbW3 | pT7-7::0.609-kb NdeI-BamHI fragment containing *rsbW3* (*MSMEG_1787*) with six His codons before its stop codons | This study |
| pT7-7rsfA | pT7-7::0.453-kb NdeI-HindIII fragment containing *rsfA* (*MSMEG_1786*) with six His codons before its stop codons | This study |
| pT7-7rsfB | pT7-7::0.384-kb NdeI-HindIII fragment containing *rsfB* (*MSMEG_6127*) with six His codons before its stop codons | This study |
| pT7-7rsfBS63A | pT7-7rsfB in which the codon for Ser-63 (TCC) is replaced with GCC | This study |
| pT7-7rsfBS63E | pT7-7rsfB in which the codon for Ser-63 (TCC) is replaced with GAG | This study |
| pMHRsbW1 | pMH201::0.454-kb NdeI-ClaI fragment containing C-terminally His_6_-tagged *rsbW1* | This study |
| pMHRsbW2 | pMH201::0.479-kb NdeI-ClaI fragment containing C-terminally His_6_-tagged *rsbW2* | This study |
| pMHRsbW3 | pMH201::0.644-kb NdeI-ClaI fragment containing C-terminally His_6_-tagged *rsbW3* | This study |
| pMHRsfA | pMH201::0.456-kb NdeI-ClaI fragment containing C-terminally His_6_-tagged *rsfA* | This study |
| pMHRsfB | pMH201::0.387-kb NdeI-ClaI fragment containing C-terminally His_6_-tagged *rsfB* | This study |
| pMHRsfBS63E | pMH201::0.387-kb NdeI-ClaI fragment from pT7-7rsfBS63E | This study |
| pKOTsΔrsfA | pKOTs::0.747-kb HindIII-NotI fragment containing Δ*rsfA* | This study |
| pKOTsΔrsfB | pKOTs::0.732-kb NotI-HindIII fragment containing Δ*rsfB* | This study |
| pKOTsΔrsbW1 | pKOTs::1.104-kb NotI-HindIII fragment containing Δ*rsbW1* | This study |
| pKOTsΔrsbW2 | pKOTs::0.750-kb NotI-HindIII fragment containing Δ*rsbW2* | This study |
| pKOTsΔrsbW3 | pKOTs::0.830-kb NotI-HindIII fragment containing Δ*rsbW3* | This study |
| pKOTsΔ5437 | pKOTs::0.825-kb HindIII-NotI fragment containing Δ*5437* | This study |
| pKOTsΔsigF | pKOTs::0.801-kb NotI-HindIII fragment containing Δ*sigF* | Bong et al., 2019 |
| pMVRsfB | pMV306::0.953-kb XbaI-HindIII fragment containing *rsfB* | This study |
| pMVRsfBT10A | pMVRsfB in which the codon for Thr-10 (ACG) is replaced with GCG | This study |
| pMVRsfBT20A | pMVRsfB in which the codon for Thr-20 (ACT) is replaced with GCG | This study |
| pMVRsfBT25A | pMVRsfB in which the codon for Thr-25 (ACC) is replaced with GCC | This study |
| pMVRsfBT27A | pMVRsfB in which the codon for Thr-27 (ACG) is replaced with GCG | This study |
| pMVRsfBT32A | pMVRsfB in which the codon for Thr-32 (ACC) is replaced with GCC | This study |
| pMVRsfBS42A | pMVRsfB in which the codon for Ser-42 (TCC) is replaced with GCC | This study |
| pMVRsfBS63A | pMVRsfB in which the codon for Ser-63 (TCC) is replaced with GCC | This study |
| pMVRsfBS63E | pMVRsfB in which the codon for Ser-63 (TCC) is replaced with GAG | This study |
| pGBKSigF | pGBKT7::0.759-kb NdeI-BamHI fragment containing *sigF* | This study |
| pGBKRsbW3 | pGBKT7::0.591-kb NdeI-BamHI fragment containing *rsbW3* | This study |
| pPLRsbW1 | pGADT7linker::0.427-kb EcoRI-XhoI fragment containing *rsbW1* | This study |
| pPLRsbW2 | pGADT7linker::0.458-kb BamHI-XhoI fragment containing *rsbW2* | This study |
| pPLRsbW3 | pGADT7linker::0.597-kb BamHI-XhoI fragment containing *rsbW3* | This study |

*Abbreviations: Amp^r^, ampicillin resistance; Cam^r^, chloramphenicol resistance; Hyg^r^, hygromycin resistance; Km^r^, kanamycin resistance. Str^r^, streptomycin resistance Tet^r^, tetracycline resistance

**TABLE S2.** Oligonucleotides used in this study

| **Oligonucleotide** | **Nucleotide sequences* (5'→3')** | **Purpose** |
| --- | --- | --- |
| F_rsfA_mut | ATTAAGCTTCTGCCGCAACAACTTTCG | Δ*rsfA* construction |
| F_rsfA_rec | AGTTCACGGCGACCTGGATGACACGGGTGCTTCGTATCTG | Δ*rsfA* construction |
| R_rsfA_rec | CAGATACGAAGCACCCGTGTCATCCAGGTCGCCGTGAACT | Δ*rsfA* construction |
| R_rsfA_mut | AATGCGGCCGCGTCCACCACATGTTCAC | Δ*rsfA* construction |
| F_rsfB_mut | ATTGCGGCCGCGATGAAGCAGGACAG | Δ*rsfB* construction |
| F_rsfB_rec | GGAGACATCACTGTGGTGGCAGACCGCTCAAACTTGTCGG | Δ*rsfB* construction |
| R_rsfB_rec | CCGACAAGTTTGAGCGGTCTGCCACCACAGTGATGTCTCC | Δ*rsfB* construction |
| R_rsfB_mut | ATTAAGCTTCGGCCTCGGTGTCGTAATC | Δ*rsfB* construction  Δ*rsfB* complementation |
| F_rsbW1_mut | ATAGCGGCCGCAGAGGGAGTCGACGC | Δ*rsbW1* construction |
| F_rsbW1_rec | GAACACCTGCCCCTCTTCAGTCTCCAACATCGCGGCGAC | Δ*rsbW1* construction |
| R_rsbW1_rec | GTCGCCGCGATGTTGGAGACTGAAGAGGGGCAGGTGTTC | Δ*rsbW1* construction |
| R_rsbW1_mut | TATAAGCTTGGCCAGCAGACGGGAG | Δ*rsbW1* construction |
| F_rsbW2_mut | ATTAGCGGCCGCAACGGTGGACATGCTGAC | Δ*rsbW2* construction |
| F_rsbW2_rec | CATCAAGGCGGGCGTAGTTGCAGCGTCACCTCAGAAC | Δ*rsbW2* construction |
| R_rsbW2_rec | GTTCTGAGGTGACGCTGCAACTACGCCCGCCTTGATG | Δ*rsbW2* construction |
| R_rsbW2_mut | ATTAAAGCTTCGAGCACAACAAGATCAAG | Δ*rsbW2* construction |
| F_rsbW3_mut | ATTAGCGGCCGCCAGTACACGGTCGAACTTC | Δ*rsbW3* construction |
| F_rsbW3_rec | CGTTTCCAGGGCTGTTTCGCAGCCCCATCCATTTCG | Δ*rsbW3* construction |
| R_rsbW3_rec | CGAAATGGATGGGGCTGCGAAACAGCCCTGGAAACG | Δ*rsbW3* construction |
| R_rsbW3_mut | ATTAAAGCTTCCTCACCGACTATGCCC | Δ*rsbW3* construction |
| F_5437_mut | ATTAAAGCTTGTGCTCGGCCATCAACTC | Δ*5437* construction |
| F_5437_rec | TCACCCGGCGAGCACATCTCCAAAGATCCCGAGCGAC | Δ*5437* construction |
| R_5437_rec | GTCGCTCGGGATCTTTGGAGATGTGCTCGCC GGGTGA | Δ*5437* construction |
| R_5437_mut | ATTAGCGGCCGCGCGACGAGGTGTTCAACC | Δ*5437* construction |
| F_sigF_mut | AATGCGGCCGCGGTGTGTATCGGCGACAATG | Δ*sigF* construction |
| F_sigF_rec | GAACCGGTTCGACGTGGAAGCGGTGGCGACGAGGAAGCC | Δ*sigF* construction |
| R_sigF_rec | GGCTTCCTCGTCGCCACCGCTTCCACGTCGAACCGGTTC | Δ*sigF* construction |
| R_sigF_mut | ATTAAGCTTCGACCAGGGAACGTACTG | Δ*sigF* construction |
| F_1777lacZ_Xba1 | ATATCTAGACGTGATCCAGTGACATGCG | pNCII1777 construction |
| R_1777lacZ_BamH1 | ATAGGATCCATGCGGCCTTGTCGTTCGTG | pNCII1777 construction |
| F_rsfB_com_BamHI | ATAGGATCCGCGATGAAGCAGGACAG | Δ*rsfB* complementation |
| F_rsfB_T10A | GACCCGGCGAACTGCGCGGTGGAGGAACGCCGC | *rsfB* point mutation |
| R_rsfB_T10A | GCGGCGTTCCTCCACCGCGCAGTTCGCCGGGTC | *rsfB* point mutation |
| F_rsfB_T20A | CGCGTAGGAGACATCGCGGTGGTGGCGGTCACC | *rsfB* point mutation |
| R_rsfB_T20A | GGTGACCGCCACCACCGCGATGTCTCCTACGCG | *rsfB* point mutation |
| F_rsfB_T25A | ACTGTGGTGGCGGTCGCCGGAACGGTGGACATG | *rsfB* point mutation |
| R_rsfB_T25A | CATGTCCACCGTTCCGGCGACCGCCACCACAGT | *rsfB* point mutation |
| F_rsfB_T27A | GTGGCGGTCACCGGAGCGGTGGACATGCTGACC | *rsfB* point mutation |
| R_rsfB_T27A | GGTCAGCATGTCCACCGCTCCGGTGACCGCCAC | *rsfB* point mutation |
| F_rsfB_T32A | ACGGTGGACATGCTGGCCGCACCGAAGCTCGAA | *rsfB* point mutation |
| R_rsfB_T32A | TTCGAGCTTCGGTGCGGCCAGCATGTCCACCGT | *rsfB* point mutation |
| F_rsfB_S42A | GAAGACGCGATCGGTGCCGCTGCCAAGAGCGAG | *rsfB* point mutation |
| R_rsfB_S42A | CTCGCTCTTGGCAGCGGCACCGATCGCGTCTTC | *rsfB* point mutation |
| F_rsfB_S63A | GGTGGATTTCCTTGCCGCCGCGGGCATGGGCGT | *rsfB* point mutation |
| R_rsfB_S63A | ACGCCCATGCCCGCGGCGGCAAGGAAATCCACC | *rsfB* point mutation |
| F_rsfB_S63E | GTGGATTTCCTTGCCGAGGCGGGCATGGGCGTG | *rsfB* point mutation |
| R_rsfB_S63E | CACGCCCATGCCCGCCTCGGCAAGGAAATCCAC | *rsfB* point mutation |
| F_sigFover | ATACATATGACGTCGGAATACG | *sigF* overexpression |
| R_sigFover | TATGGATCCTCAGTGATGGTGATGGTGATGCTGCAGCTGGTCGCGCAG | *sigF* overexpression |
| F_rsbW1over | TATCATATGGCGGAAACACCCGCTCG | *rsbW1* overexpression |
| R_rsbW1over | TATCTGCAGTCAGTGATGGTGATGGTGATGCCGCAGCAGGCTCGCTCGCC | *rsbW1* overexpression |
| F_rsbW2over | ATTTCATATGACAGACGCAGGCGAG | *rsbW2* overexpression |
| R_rsbW2over | ATATAAGCTTTTAGTGATGGTGATGGTGATGCCGCAGTTCTGAGGT | *rsbW2* overexpression |
| F_rsbW3over | ATTACATATGTCCCTGGGCGGCAAT | *rsbW3* overexpression |
| R_rsbW3over | ATTAGGATCCCTAGTGATGGTGATGGTGATGGTCCGACACCGCGTC | *rsbW3* overexpression |
| F_rsfAover | ATTACATATGCCCACAATCAGCGTTG | *rsfA* overexpression |
| R_rsfAover | ATTAAAGCTTTCAGTGATGGTGATGGTGATGGGTGTTCTCCACCAGTTG | *rsfA* overexpression |
| F_rsfBover | ATTACATATGACGAGCCAGGACCCG | *rsfB* overexpression |
| R_rsfBover | ATTAAAGCTTTCAGTGATGGTGATGGTGATGTGTCTTCAACGACGAGAGGG | *rsfB* overexpression |
| F_sigF_NdeI | ATTACATATGACGTCGGAATACGCA | Y2H |
| R_sigF_BamHI | ATTAGGATCCCTACTGCAGCTGGTCGCG | Y2H |
| R_rsbW3_BamHI | ATTAGGATCCCTAGTC CGA CAC CGC GTC | Y2H |
| F_rsbW1_EcoRI | ATTAGAATTCATGGCGGAAACACCCGCT | Y2H |
| R_rsbW1_XhoI | ATTACTCGAGTCACCGCAGCAGGCTCGC | Y2H |
| F_rsbW2_BamHI | ATTAGGATCCATGACAGACGCAGGCGAG | Y2H |
| R_rsbW2_XhoI | ATTACTCGAGTTACCGCAGTTCTGAGGT | Y2H |
| F_rsbW3_BamHI | ATTAGGATCCCCATGTCCCTGGGCGGCAAT | Y2H |
| R_rsbW3_XhoI | ATTACTCGAGCTAGTCCGACACCGCGTC | Y2H |
| RT_16SrRNA_F | CTGGGACTGAGATACGGC | RT-PCR for 16S rRNA |
| RT_16SrRNA_R | ACAACGCTCGGACCCTAC | RT-PCR for 16S rRNA |
| RT_sigA_F | CTGGAGGCGAACCTGCGC | RT-PCR for *sigA* |
| RT_sigA_R | CTGGTCGGCCATGGCGCG | RT-PCR for *sigA* |
| RT_rsbW1_F | GAGCGGTCGGTAGAGATC | RT-PCR for *rsbW1* |
| RT_rsbW1_R | GACATTGTCGCCGATACACAC | RT-PCR for *rsbW1* |
| RT_rsbW2_F | CACGCATTTCACGCTCGACCCG | RT-PCR for *rsbW2* |
| RT_rsbW2_R | CGCCTATCCGCCATGTGCC | RT-PCR for *rsbW2* |
| RT_rsbW3_F | ATGGGGCTGCCGTCATCG | RT-PCR for *rsbW3* |
| RT_rsbW3_R | ACGTCGCTGTCCAAACCC | RT-PCR for *rsbW3* |
| RT_rsfA_F | ATGCCCACAATCAGCGTTGC | RT-PCR for *rsfA* |
| RT_rsfA_R | ACACGCGGCTCCGAAGAATG | RT-PCR for *rsfA* |
| RT_rsfB_F | GTTGCGCCGCGAATTCTC | RT-PCR for *rsfB* |
| RT_rsfB_R | CTATCCGCCATGTGCCCTC | RT-PCR for *rsfB* |
| RT_1777_F | GCTGAAAGACACTGCCAAC | RT-PCR for *MSMEG_1777* |
| RT_1777_R | CATTCCATTGACGTTCCTTG | RT-PCR for *MSMEG_1777* |
| RT_1782_F | CCTCAACGATCAGCACAACG | RT-PCR for *MSMEG_1782* |
| RT_1782_R | ACCAACGATGCGGTATGGGTG | RT-PCR for *MSMEG_1782* |
| Left_pNC_BamHI | GAGCCATGGTGAATCGAGCTGTCGACATCGATAAG | pNCII construction |
| Right_pNC_BamHI | CTTATCGATGTCGACAGCTCGATTCACCATGGCTC | pNCII construction |

**TABLE S3.** Summarized statistics of RNA sequencing alignment

|  | **WT_1** | **WT_2** | **WT_3** | **Δ*aa_3_*_1** | **Δ*aa_3_*_2** | **Δ*aa_3_*_3** |
| --- | --- | --- | --- | --- | --- | --- |
| Read length (bp) | 101 | 101 | 101 | 101 | 101 | 101 |
| Total No. of PE^a^ reads | 32,090,124 | 39,369,322 | 33,922,020 | 38,066,636 | 45,159,958 | 39,096,948 |
| No. of processed reads | 16,045,062 | 19,684,661 | 16,961,010 | 19,033,318 | 22,579,979 | 19,548,474 |
| No. of mapped reads | 14,753,758 | 18,288,726 | 15,536,559 | 17,553,432 | 20,575,530 | 17,739,562 |
| No. of failed to align reads | 672,497 | 683,900 | 636,318 | 595,005 | 869,849 | 758,051 |
| No. of suppressed reads by multiple mapping | 618,807 | 712,035 | 788,133 | 884,881 | 1,134,600 | 1,050,861 |

^a^ paired-end

**TABLE S4.** The genes that are induced in the Δ*aa_3_* mutant of *M. smegmatis* by more than 4-fold relative to the WT strain and belong to the SigF regulon

| **Locus tag** | **Description** | **log_2_FC** | ***P*-value** | **Promoter** |
| --- | --- | --- | --- | --- |
| MSMEG_0266 | Arginine decarboxylase | 2.5 | 0.035 | GTCG-N_17_-GGGAT |
| MSMEG_0267 | Alpha/beta hydrolase | 3.4 | 0.022 | GTTT-N_15_-GGGTA |
| MSMEG_0451 | Hypothetical protein | 3.3 | 0.015 | GTTC-N_19_-GGGCC |
| MSMEG_0672 | GAF domain-containing protein | 3.0 | 0.024 | GTTT-N_15_-GGGTA |
| MSMEG_0685 | Xanthine dehydrogenase family protein subunit M | 3.2 | 0.027 | GTTG-N_15_-GGGTA |
| MSMEG_0686 | 2Fe-2S iron-sulfur cluster binding domain-containing protein | 3.4 | 0.026 | GTTG-N_15_-GGGTA |
| MSMEG_0697 | Phage holin family protein | 2.6 | 0.031 | GTTT-N_16_-GGGAA |
| MSMEG_1076 | Hypothetical protein | 3.1 | 0.042 | GTTT-N_16_-GGGTA |
| MSMEG_1112 | Aconitate hydratase | 2.8 | 0.026 | CGTT-N_16_-GGGAA |
| MSMEG_1131 | Tryptophan-rich sensory protein | 2.7 | 0.019 | GTGT-N_16_-GGGTA |
| MSMEG_1758 | Hypothetical protein | 3.5 | 0.017 | GTTT-N_16_-GGGTA |
| MSMEG_1766 | Hypothetical protein | 3.0 | 0.028 | GTTT-N_16_-GGGAA |
| MSMEG_1767 | Hypothetical protein | 3.0 | 0.030 | GTTT-N_16_-GGGAA |
| MSMEG_1768 | Flavodoxin | 2.8 | 0.039 | GTTT-N_16_-GGGAA |
| MSMEG_1771 | Methyltransferase | 3.4 | 0.029 | GTTT-N_15_-GGGTA |
| MSMEG_1774 | DUF4383 domain-containing protein | 3.7 | 0.021 | GTTT-N_16_-GGGTA |
| MSMEG_1775 | Cytochrome P450 monooxygenase | 2.7 | 0.025 | GTTT-N_15_-GGGTA |
| MSMEG_1777 | UsfY | 3.5 | 0.014 | GTTT-N_16_-GGGTA |
| MSMEG_1778 | Hypothetical protein | 3.1 | 0.046 | GTTT-N_15_-GGGTA |
| MSMEG_1782 | SDR family oxidoreductase | 3.4 | 0.025 | GTTT-N_15_-GGGTA |
| MSMEG_1783 | Hypothetical protein | 3.4 | 0.022 | GTGT-N_16_-GGGTA |
| MSMEG_1787 | RsbW3 | 3.0 | 0.025 | GTTT-N_17_-GGGTA |
| MSMEG_1788 | Hypothetical protein | 3.5 | 0.032 | GGTT-N_15_-GGGCA |
| MSMEG_1792 | Hypothetical protein | 3.0 | 0.022 | GGGT-N_14_-GGGCA |
| MSMEG_1794 | LLM class F420-dependent oxidoreductase | 3.2 | 0.014 | GTGT-N_17_-GGGTA |
| MSMEG_1802 | ChaB protein | 3.1 | 0.021 | GTTT-N_16_-GGGCA |
| MSMEG_1950 | Hypothetical protein | 3.8 | 0.014 | GTCG-N_16_-GGGCA |
| MSMEG_1951 | Hypothetical protein | 3.5 | 0.028 | GTCG-N_16_-GGGCA |
| MSMEG_2347 | Phytoene dehydrogenase | 2.9 | 0.048 | GTTT-N_16_-GGGTA |
| MSMEG_2376 | Hypothetical protein | 3.1 | 0.024 | GTTC-N_19_-GGGCC |
| MSMEG_2415 | Hemerythrin domain-containing protein | 3.1 | 0.030 | GTGT-N_15_-GGGTA |
| MSMEG_2837 | Molybdopterin-dependent oxidoreductase | 2.5 | 0.031 | GTTT-N_16_-GGGTA |
| MSMEG_2958 | Hypothetical protein | 3.1 | 0.015 | GTTC-N_15_-GGGTA |
| MSMEG_3022 | GlsB/YeaQ/YmgE family stress response membrane protein | 3.5 | 0.034 | GTTT-N_16_-GGGTA |
| MSMEG_3255 | DoxX subfamily protein | 3.1 | 0.018 | GTTT-N_15_-GGGAA |
| MSMEG_3273 | M20/M25/M40 family metallo-hydrolase | 2.8 | 0.028 | GCTT-N_16_-GGGCC |
| MSMEG_3289 | Gp61 protein | 3.6 | 0.027 | GTTT-N_15_-GGGTA |
| MSMEG_3304 | Succinate semialdehyde dehydrogenase | 2.5 | 0.042 | GTGT-N_15_-GGGTA |
| MSMEG_3419 | Hypothetical protein | 3.6 | 0.023 | GTCG-N_14_-GGGTA |
| MSMEG_3536 | MFS transporter | 2.4 | 0.028 | GTGG-N_16_-GGGTA |
| MSMEG_4993 | Hypothetical protein | 2.8 | 0.022 | GTGT-N_19_-GGGCA |
| MSMEG_5188 | CPBP family intramembrane metalloprotease | 2.3 | 0.044 | GGTT-N_16_-GGGTA |
| MSMEG_5189 | NAD(P)H-binding protein | 2.6 | 0.018 | GGTT-N_16_-GGGTA |
| MSMEG_5342 | Hypothetical protein | 3.1 | 0.026 | GTTT-N_16_-GGCTA |
| MSMEG_5401 | SRPBCC family protein | 2.3 | 0.037 | GTTT-N_15_-GGGTA |
| MSMEG_5402 | SDR family NAD(P)-dependent oxidoreductase | 3.3 | 0.015 | GTTT-N_15_-GGGTA |
| MSMEG_5543 | Hypothetical protein | 3.0 | 0.028 | GTTT-N_17_-GGGTA |
| MSMEG_5590 | Glutamate-cysteine ligase | 3.3 | 0.014 | GTTT-N_15_-GGGCA |
| MSMEG_5605 | Cytochrome bd ubiquinol oxidase subunit I | 2.2 | 0.037 | GGTG-N_19_-GGGAA |
| MSMEG_6210 | Hypothetical protein | 2.4 | 0.015 | GTTC-N_14_-GGGTG |
| MSMEG_6213 | Manganese containing catalase | 3.0 | 0.035 | GTTT-N_15_-GGGTA |
| MSMEG_6354 | Cutinase family protein | 2.7 | 0.026 | GTTT-N_16_-GGGAA |
| MSMEG_6467 | Starvation-induced DNA protecting protein | 3.2 | 0.035 | GTTC-N_16_-GGGCA |
| MSMEG_6612 | AAA domain-containing protein | 3.1 | 0.027 | GTTC-N_14_-GGGCA |
| MSMEG_6615 | Dithiol-disulfide isomerase | 3.1 | 0.050 | GTTT-N_15_-GGGTA |
| MSMEG_6616 | S-(hydroxymethyl)glutathione dehydrogenase | 3.2 | 0.026 | GTTT-N_15_-GGGTA |
| MSMEG_6665 | Glutathione-dependent formaldehyde dehydrogenase | 3.3 | 0.017 | GTTT-N_15_-GGGAA |
| MSMEG_6667 | DUF2243 domain-containing protein | 3.0 | 0.036 | GTTT-N_15_-GGGAA |
| MSMEG_6768 | Extracellular solute-binding protein | 3.1 | 0.030 | GCTT-N_16_-GGGTA |
| MSMEG_6812 | Pseudogene | 2.2 | 0.033 | GGTT-N_14_-GGGGA |
| MSMEG_6819 | Pseudogene | 3.0 | 0.017 | GGTG-N_19_-GGGCG |

The fold change (FC) of gene expression indicates the ratio of the transcript level of a gene in the Δ*aa_3_* strain to that in the WT strain. The downregulated genes (log_2_FC < -2) in a *sigF* mutant strain grown to stationary phase relative to the WT strain grown under the same conditions were regarded as the gene belonging to the SigF regulon (Singh et al., 2015).

**REFERENCES**

Bong, H.J., Ko, E.M., Song, S.Y., Ko, I.J., and Oh, J.I. (2019). Tripartite Regulation of the *glpFKD* Operon Involved in Glycerol Catabolism by GylR, Crp, and SigF in *Mycobacterium smegmatis*. *J. Bacteriol.* 201. doi:10.1128/JB.00511-19

Brown, A.K., Bhatt, A., Singh, A., Saparia, E., Evans, A.F., and Besra, G.S. (2007). Identification of the dehydratase component of the mycobacterial mycolic acid-synthesizing fatty acid synthase-II complex. *Microbiology* 153**,** 4166-4173. doi:10.1099/mic.0.2007/012419-0

James, P., Halladay, J., and Craig, E.A. (1996). Genomic libraries and a host strain designed for highly efficient two-hybrid selection in yeast. *Genetics* 144**,** 1425-1436.

Jeong, J.A., Lee, H.N., Ko, I.-J., and Oh, J.-I. (2013). Development of new vector systems as genetic tools applicable to mycobacteria. *J. Life Sci.* 23**,** 290-298.

Jeong, J.A., Park, S.W., Yoon, D., Kim, S., Kang, H.Y., and Oh, J.I. (2018). Roles of alanine dehydrogenase and Induction of Its gene in *Mycobacterium smegmatis* under respiration-inhibitory conditions. *J. Bacteriol.* 200**,** e00152-00118. doi:10.1128/JB.00152-18

Jessee, J. (1986). New subcloning efficiency competent cells: >1x10^6^ transformants/mg. *Focus* 8**,** 1146-1157.

Kang, C.M., Abbott, D.W., Park, S.T., Dascher, C.C., Cantley, L.C., and Husson, R.N. (2005). The *Mycobacterium tuberculosis* serine/threonine kinases PknA and PknB: substrate identification and regulation of cell shape. *Genes Dev.* 19**,** 1692-1704. doi:10.1101/gad.1311105

Lee, H.N., Ji, C.J., Lee, H.H., Park, J., Seo, Y.S., Lee, J.W., and Oh, J.I. (2018). Roles of three FurA paralogs in the regulation of genes pertaining to peroxide defense in *Mycobacterium smegmatis* mc^2^ 155. *Mol. Microbiol.* 108**,** 661-682. doi:10.1111/mmi.13956

Lee, H.N., Jung, K.E., Ko, I.J., Baik, H.S., and Oh, J.I. (2012). Protein-protein interactions between histidine kinases and response regulators of *Mycobacterium tuberculosis* H37Rv. *J. Microbiol.* 50**,** 270-277. doi:10.1007/s12275-012-2050-4

Louvet, O., Doignon, F., and Crouzet, M. (1997). Stable DNA-binding yeast vector allowing high-bait expression for use in the two-hybrid system. *Biotechniques* 23**,** 816-818, 820. doi:10.2144/97235bm11

Oh, J.I., Park, S.J., Shin, S.J., Ko, I.J., Han, S.J., Park, S.W., Song, T., and Kim, Y.M. (2010). Identification of trans- and cis-control elements involved in regulation of the carbon monoxide dehydrogenase genes in Mycobacterium sp. strain JC1 DSM 3803. *J. Bacteriol.* 192**,** 3925-3933. doi:10.1128/JB.00286-10

Singh, A.K., Dutta, D., Singh, V., Srivastava, V., Biswas, R.K., and Singh, B.N. (2015). Characterization of *Mycobacterium smegmatis* *sigF* mutant and its regulon: overexpression of SigF antagonist (MSMEG_1803) in *M. smegmatis* mimics *sigF* mutant phenotype, loss of pigmentation, and sensitivity to oxidative stress. *Microbiology* 4**,** 896-916. doi:10.1002/mbo3.288

Stover, C., De La Cruz, V., Fuerst, T., Burlein, J., Benson, L., Bennett, L., Bansal, G., Young, J., and Lee, M.-H. (1991). New use of BCG for recombinant vaccines. *Nature* 351**,** 456–460.

Tabor, S. and Richardson, C.C. (1985). A bacteriophage T7 RNA polymerase/promoter system for controlled exclusive expression of specific genes. *Proc. Natl. Acad. Sci. U.S.A.* 82**,** 1074-1078. doi:10.1073/pnas.82.4.1074
